# Stem Cell Extracellular Vesicles as Anti-SARS-CoV-2 Immunomodulatory Therapeutics: A Systematic Review of Clinical and Preclinical Studies

**DOI:** 10.1007/s12015-023-10675-2

**Published:** 2024-02-23

**Authors:** Sarah Hamdy Ahmed, Mohamed Atef AlMoslemany, Kenneth Whitaker Witwer, Ahmed Gamal Tehamy, Nagwa El-Badri

**Affiliations:** 1 Center of Excellence for Stem Cells and Regenerative Medicine (CESC), Zewail City of Science and Technology, October Gardens, Giza, 6th of October City, 12582 Egypt; 2 Biotechnology/Biomolecular Chemistry Department, Faculty of Science, Cairo University, Giza, 12613 Egypt; 3 Department of Molecular and Comparative Pathobiology, The Johns Hopkins University School of Medicine, Baltimore, MD USA; 4 Department of Neurology and Neurosurgery, The Johns Hopkins University School of Medicine, Baltimore, MD USA; 5 Richman Family Precision Medicine Center of Excellence in Alzheimer’s Disease, The Johns Hopkins University School of Medicine, Baltimore, MD USA

**Keywords:** Extracellular Vesicles, Exosomes, COVID-19, SARS-CoV-2, Acute respiratory distress syndrome, ARDS, Pneumonia, Stem cells, Regenerative medicine, microRNAs

## Abstract

**Background:**

COVID-19 rapidly escalated into a worldwide pandemic with elevated infectivity even from asymptomatic patients. Complications can lead to severe pneumonia and acute respiratory distress syndrome (ARDS), which are the main contributors to death. Because of their regenerative and immunomodulatory capacities, stem cells and their derived extracellular vesicles (EVs) are perceived as promising therapies against severe pulmonary conditions, including those associated with COVID-19. Herein, we evaluate the safety and efficacy of stem cell EVs in treating COVID-19 and complicating pneumonia, acute lung injury, and ARDS. We also cover relevant preclinical studies to recapitulate the current progress in stem cell EV-based therapy.

**Methods:**

Using PubMed, Cochrane Central Register of Controlled Trials, Scopus, and Web of Science, we searched for all English-language published studies (2000–2023) that used stem cell EVs as a therapy for COVID-19, ARDS, or pneumonia. The risk of bias (ROB) was assessed for all studies.

**Results:**

Forty-eight studies met our inclusion criteria. Various-sized EVs derived from different types of stem cells were reported as a potentially safe and effective therapy to attenuate the cytokine storm induced by COVID-19. EVs alleviated inflammation and regenerated the alveolar epithelium by decreasing apoptosis, proinflammatory cytokines, neutrophil infiltration, and M2 macrophage polarization. They also prevented fibrin production and promoted the production of anti-inflammatory cytokines and endothelial cell junction proteins.

**Conclusion:**

Similar to their parental cells, stem cell EVs mediate lung tissue regeneration by targeting multiple pathways and thus hold promise in promoting the recovery of COVID-19 patients and improving the survival rate of severely affected patients.

**Graphical Abstract:**

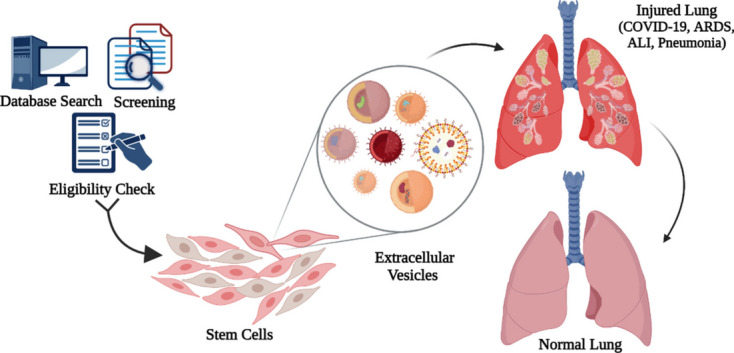

**Supplementary Information:**

The online version contains supplementary material available at 10.1007/s12015-023-10675-2.

## Introduction

 COVID-19 was declared a global pandemic on the 11th of March 2020 by the World Health Organization (WHO) [1]. Caused by severe acute respiratory syndrome coronavirus-2 (SARS-CoV-2), COVID-19 has resulted in mortality rates exceeding 5.5 million and 272 million reported cases within two years [2]. As a positive-sense single-stranded RNA virus, SARS-CoV-2 rapidly developed various mutations unleashing multiple variants of concern, including alpha (B.1.1.7), beta (B.1.351), gamma (P.1), delta (B.1.617.2) [1], and Omicron (B.1.1.529) [2]. In humans, this virus is transmitted via respiratory droplets and affects patients of different ages and sexes with fluctuating virulence levels [3].

Upon infection with SARS-CoV-2, the patient’s immune system induces an inflammatory “cytokine storm” to defeat the virus. This response can also result in damage and aggravate other conditions, including interstitial pneumonia, acute respiratory distress syndrome (ARDS), multiple organ failure, or even death, depending on many factors, including the strength of the patient’s immune system [4, 5]. In an early study, ARDS was reported in 42% of COVID-19 patients, and 61–81% of the total cohort needed intensive care due to severe hypoxemia that required mechanical ventilation [6]. Affected lung tissue displayed endothelial damage with severe inflammation, poor pulmonary oxygenation, increased vascular permeability, and pulmonary interstitial fibrosis [7]. ARDS is thus considered more severe in COVID-19 patients and even results in post-COVID-19 pulmonary fibrosis in some survivors [8].

Multiple treatments for COVID-19 have been implemented or proposed. Some treatments, such as repurposed anti-malarial and anti-viral drugs, may improve recovery and survival rates but do not regenerate damaged lung tissue [9]. In contrast, stem cell therapy was proposed as a COVID-19 approach specifically because of promising regenerative capacities for a plethora of cardiovascular [10], degenerative [11], and lung diseases [12]. The therapeutic effects of stem cells are attributed to anti-inflammatory, immunomodulatory, regenerative, pro-angiogenic, and anti-fibrotic properties, along with a vast variety of potential sources [13]. Stem cells may be especially attractive as COVID-19 treatments since early passages do not display the SARS-CoV-2 receptor (angiotensin-converting enzyme 2 (ACE2)), rendering them resistant to infection [14]. Different stem cells and their secretomes have thus been tested in preclinical and clinical settings to combat COVID-19 complications [15, 16]. For example, umbilical cord, adipose and bone marrow-derived mesenchymal stem cells have successfully ameliorated the cytokine storm by regulating immune cells such as macrophages, neutrophils, B and T cells, DCs, and natural killer cells [17–20].

Stem cell therapy faces the challenges of finding proper tissue matching, the use of immune suppressive regimens, and the complications of graft rejection or graft vs. host disease. Extracellular vesicles (EVs) may thus present a viable alternative, as they provide many of the stem cell regenerative properties and avoid the complications of whole-cell therapy. EVs are small lipid bilayer nanovesicles of different sizes and origins that are released by live cells and possess the same immunomodulatory and regenerative properties as their parental cells [21, 22]. Since cell-based therapies also face application hurdles, including large-scale production and the limitations of reconstituting cryopreserved cells, EVs are shifting regenerative medicine from cellular to acellular therapy [23, 24]. In addition to advantages such as low immunogenic and teratogenic properties, EVs have been reported to trigger anti-inflammatory cytokine release and reduce inflammation [24, 25]. In different lung injury models, EVs are thought to function by shuttling microRNA, mRNA, DNA, proteins, and metabolites to and/or into recipient cells in injured tissue, thereby promoting repair and regeneration [26]. At the time of this writing, at least 28 clinical trials had been registered worldwide to investigate the safety and/or efficacy of stem cell EVs for ARDS and/or pneumonia in patients critically ill with COVID-19 (ClinicalTrials.gov, Chinese Clinical Trial Register (ChiCTR), IRCT, ISRCTN Registry, EU Clinical Trials Register, last accessed: 9th of September 2022). Amidst this growing interest in EV therapies, we aim in this systematic review to assess the immunomodulatory effects and efficacy of stem cell EVs in treating severe pulmonary conditions associated with COVID-19, such as acute lung injury (ALI), ARDS, and severe pneumonia in humans. The review also covers preclinical studies in induced animal models and recapitulates the current progress in stem cell EV-based therapy.

## Methods

### Literature Search Strategy

A defined literature search was conducted by three authors independently using the following databases: PubMed, Scopus, Web of Science, and Cochrane Central Register of Controlled Trials. The following MeSH headings and keywords were used: “extracellular vesicles”, “exosomes”, “microvesicles”, “COVID-19”, “SARS-CoV-2”, “coronavirus”, “acute respiratory distress syndrome”, “acute lung injury”, “pneumonia”, and “stem cells”. Studies published from 2000 until May 31, 2023, were included. This review was reported using the Preferred Reporting Items for Systematic Reviews and Meta-analysis (PRISMA) statement [27] and was registered on the international prospective register of systematic reviews (PROSPERO; CRD42022335053 and CRD42022336501) [28].

### Inclusion Criteria

We included all in vitro studies, preclinical in vivo animal studies, and clinical interventional studies of stem cell EVs of various designations (extracellular vesicles, microvesicles, and exosomes) from any tissue source (bone marrow, adipose, umbilical cord, dental pulp, placenta, etc.) used as an intervention to treat COVID-19, ARDS and/or lung injury. We included studies that used syngeneic, allogeneic, or xenogeneic cells as the secretome source. Studies that were fully accessible and published in English were included. The PICO search strategy is provided as supplemental file 1.

### Exclusion Criteria

We excluded studies that (1) administered only non-stem cell-based therapeutics to treat COVID-19, such as cell therapy using somatic cells other than stem cells, antiviral, immunomodulatory, and anti-cytokine drugs or a combination thereof; (2) did not report EV isolation methods; (3) were conducted in silico only; (4) were on pulmonary fibrosis, asthma or other respiratory conditions that were not directly related to COVID-19; (5) were review articles, meta-analyses, comments, notes, book chapters or surveys and theses, conference proceedings and editorials; and (6) used EVs to treat organs other than the lungs.

### Study Selection and Data Extraction

Using the inclusion and exclusion criteria, the title and abstract were initially screened independently by SA, MA, and AG via Rayyan.ai (https://rayyan.qcri.org/), an online platform for the study selection process. Differences in opinion or discrepancies were resolved by discussion and consultation with the NB. All duplicated studies were checked and removed before the study selection process. Full-text articles were retrieved by three authors independently to assess the final eligibility. Data extraction and subsequent full-text review were performed using an Excel data extraction form to search for data in the Results and Method sections as well as in tables, graphs, and figures. Cross-checking of the data retrieved for each article was performed by the other authors. Specific data extracted from studies included study characteristics (e.g., lead author, year of publication, country), study design, and intervention characteristics (e.g., sample size, source of stem cell, reported size and type of EV, EV separation and characterization methods, mode of administration, EV dosage, and time of assessment). All data about the primary and secondary outcomes (e.g., survival rate, lung injury score, oxygenation level, days in ICU, inflammatory cytokine concentrations, CT, laboratory and radiologic findings, and adverse events) were also recorded.

### Outcome Measures

Patient survival rate and measures of efficacy of EVs in targeting COVID-19 severity according to the World Health Organization Ordinal Scale for Clinical Improvement (WHO-OSCI) were included as primary outcome measures [29]. These included oxygenation levels (e.g., PaO2/FiO2 ratio), anti-inflammatory cytokines (IL-10, transforming growth factor [TGF]-b, etc.), inflammatory markers (D-dimer, C-reactive protein, ferritin, etc.), circulating levels of immune cells (lymphocytes, neutrophils, macrophages, regulatory dendritic cells, NK cells, etc.), proinflammatory cytokines (IL-6, IL-8, tumor necrosis factor [TNF]α, interferon [IFN]γ, etc.), organ failure assessment score (e.g., Sequential Organ Failure Assessment [SOFA]), and adverse events following EV administration (tumorigenesis, thromboembolism, etc.). Importantly, for stem cell EVs of different designations, we included EV source, EV separation and characterization method, biological effects, and the route, formulation, and dosage of their administration.

### Quality Assessment

We used the Cochrane Bias Risk Assessment tools to assess the quality of the included studies, where the risk of bias was based on the following criteria: random sequence generation (selection bias), blinding of outcome assessment (detection bias), incomplete outcome data (attrition bias), allocation concealment (selection bias), blinding of participants and researchers (performance bias), selective reporting (reporting bias) and other bias. The RoB-2 tool for randomized studies was used to assess the risk of bias for the interventional controlled studies [30]. The ROBINS-I tool for nonrandomized studies was used to assess the risk of bias for the interventional controlled studies [31]. Additionally, we used the systematic review center for laboratory animal experimentation (SYRCLE) risk of bias tool to evaluate the risk of bias in preclinical studies [32]. The Confidence in the Evidence from the Reviews of Qualitative research (CERQual) tool was used to assess the evidence quality of each outcome in the systematic review [33]. A PRISMA figure following the PRISMA checklist criteria was created [27].

## Results

### Literature Search

**Fig. 1:**
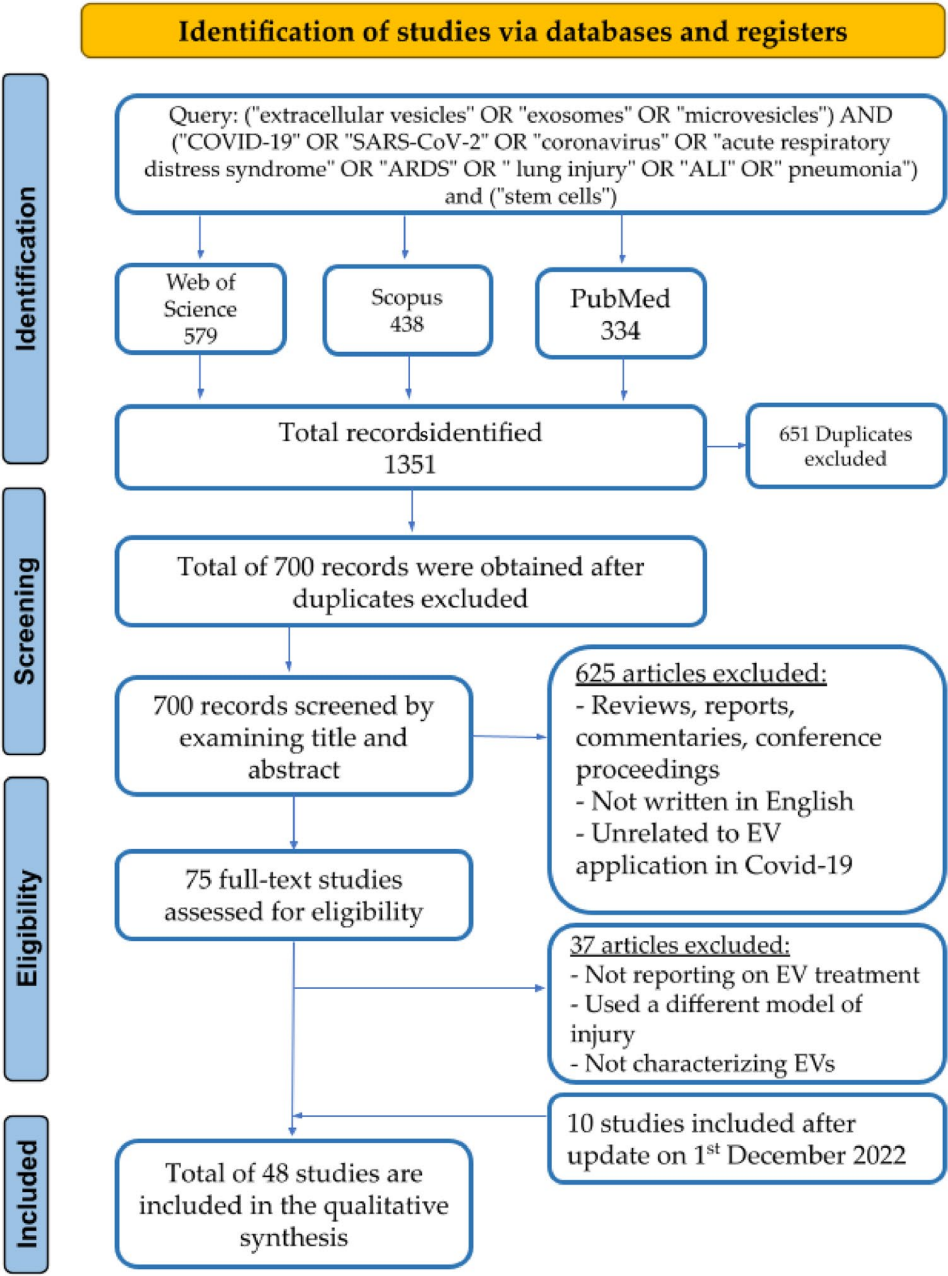
Study flow diagram

A search on PubMed, Web of Science, Scopus, and Cochrane Central Register of Controlled Trials retrieved a total of 1351 studies. After removing duplicate publications, the title and/or abstract of 700 studies were screened to include articles that assessed the efficacy and/or safety of stem cell EVs in combating COVID-19 or severe pulmonary conditions such as ARDS, ALI, and pneumonia. We excluded 625 studies that were review articles, meta-analyses, comments, news, book chapters, surveys, theses, conference papers, and/or editorials. These studies were also excluded because they did not match the outcome of interest (e.g., lung injury models not relevant to COVID-19, such as bronchopulmonary dysplasia, cystic fibrosis, and asthma) or the treatment criteria (e.g., stem cell EVs employed as biomarkers and not for treatment) or were reported in languages other than English. Thus, the remaining 75 articles were assessed for eligibility via full-text screening. Thirty-seven studies that did not characterize EVs by size and/or at least one protein marker were excluded, and 48 studies were eventually retained for final analysis (Fig. 1) [34–81]. The selected studies were published between 2000 and 2023 and included in vitro, ex vivo, in vivo, and human subjects and/ or human-origin materials. These studies targeted COVID-19, ALI/ARDS, pneumonia, or allergic airway inflammation. A list of excluded articles is provided in supplemental file 2.

### Study Characteristics

**Fig. 2:**
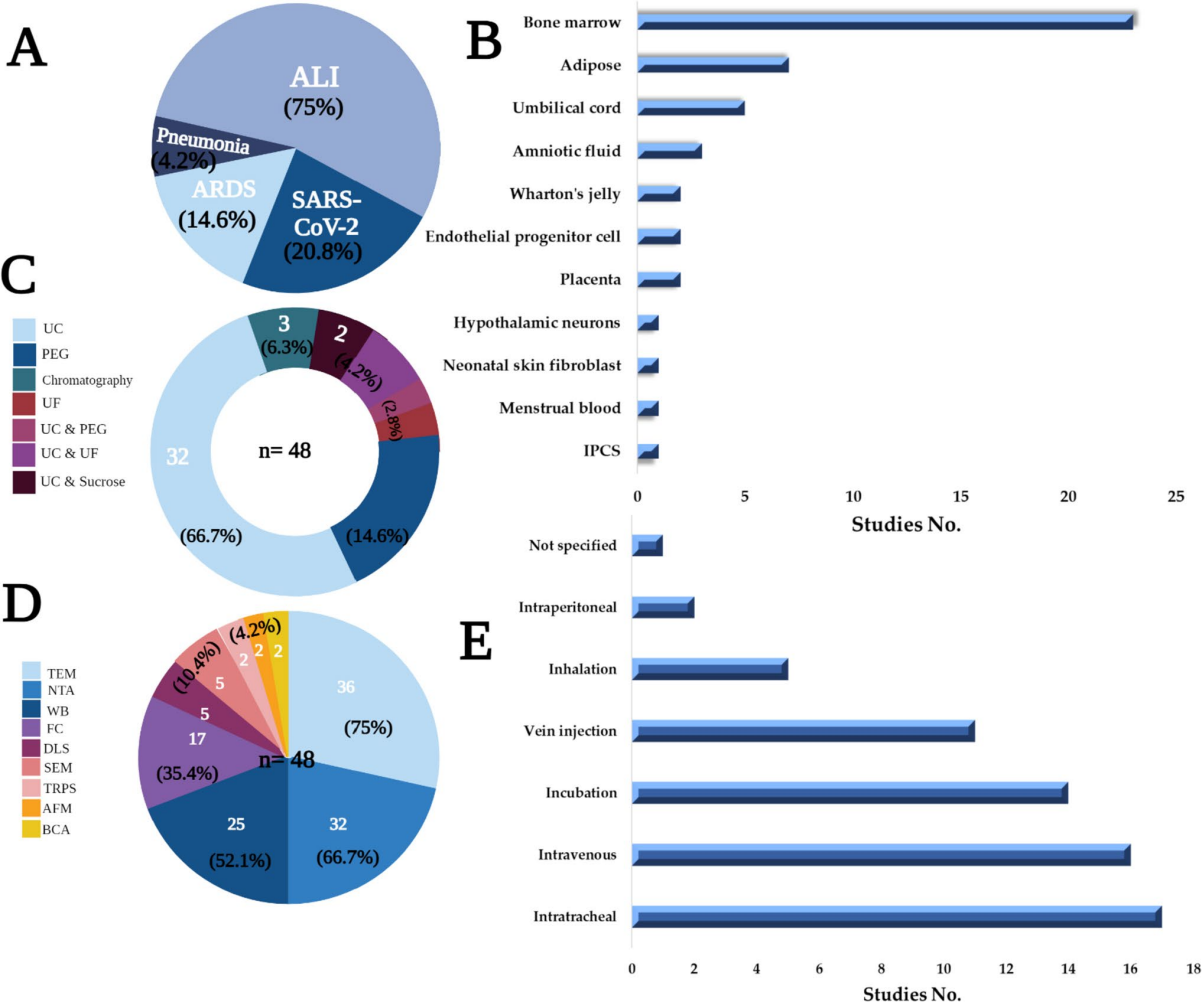
An overview of studies characteristics. **A** Ratios of pulmonary diseases including ARDS, COVID-19, ALI, and pneumonia. **B** Stem cells sources to separate EVs with number of studies using each source. **C** Percentages of different EV separation methods. **D** Percentages of characterization procedures used for EV identification with number of studies. **E** EV administration routes reflected by the number of studies for each route. UC: ultracentrifugation; UF: ultrafiltration; TEM: transmission electron microscopy; NTA: nano tracking analysis; WB: western blot; FC: flowcytometry; DLS: dynamic light scattering; SEM: scanning electron microscopy; TRPS; tunnel resistive pulse sensing; AFM: atomic force microscopy; BCA: bicinchoninic acid assay

 The 48 studies remaining after the application of the inclusion and exclusion criteria included seven studies of human patients or tissues. The 48 studies were targeting ALI (75%), SARS-CoV-2 (20.8%), ARDS (14.6%), and pneumonia (4.2%) (Fig. 2A). Four studies targeted severely ill COVID-19 patients. Two studies examined mild to moderate COVID-19 patients or long-haul patients via two FDA-approved (Exoflo and Zofin) treatments. One study used an *Escherichia coli*-induced model of severe pneumonia in ex vivo perfused human lungs. To model COVID-19 or ALI/ARDS in experimental animals, inducing agents including lipopolysaccharide (LPS), *Escherichia coli*, endotoxin, *Pseudomonas aeruginosa*, histones, bleomycin, burn injury, influenza virus injection, ovalbumin, cytokine exposure, trauma or mechanical ventilation injury. These studies primarily evaluated the therapeutic efficacy and safety of EVs derived from various sources as a cell-free therapy for recovery from lung injury. The major sources for these EVs were MSCs derived from human or animal bone marrow, adipose tissue, umbilical cords, amniotic fluid, and Wharton’s jelly. However, other EV sources, such as placenta, endothelial progenitor cells, neuronal stem cells, human neonatal fibroblasts, menstrual blood, and IPCS were included (Fig. 2B). All but one in vivo study (of commercial pigs as a large animal model) was carried out in mice or rats. Table 1 shows the reported characteristics of stem cell EVs in all selected studies.

**Table 1:** Characteristics of stem cell EVs employed in included studies

Study	EV separation	EV characterization	Reported nomenclature and size	Treatment (Method/Dose)	Time of administration	Reference
Zhu et al.	UC and 12% PEG	TEM, NTA, WB	EVs (50–400 nm)	Inhalation 5 doses: 6 ml diluted with normal saline (1.0 × 10^9^ haMSCExos per patient)	On days 1, 2, 3, 4 and 5	[34]
Mitrani et al.	UC	NTA, TEM, MACSPlex exosome kit	Zofin: EVs and Exosomes (90.2 nm)	Intravenous 3 doses: 1 mL diluted in 100 mL of normal saline	On days 0, 4, and 8	[35]
Mitrani et al.	UC	NTA, TEM, MACSPlex exosome kit	Zofin: EVs and Exosomes (90.2 nm)	Intravenous 4 doses: 1 mL diluted in 100 mL of normal saline	On days 0, 4, 6, and 8	[36]
Bellio et al.	UC	NTA, TEM, MACSPlex exosome kit	Zofin: EVs (99.7 nm)	Intravenous 3 doses: 1 mL diluted in 100 mL of normal saline	On days 0, 4, and 8	[37]
Sengupta et al.	UC	SP-IRIS, NTA	ExoFlo: EVs and Exosomes (30–150 nm)	Intravenous 1 dose: 15 ml of ExoFlo was added to 100 mL of normal saline	After baseline testing	[38]
FKazerooni et al.	Centrifugation and filtration	Flow cytometry	EVs	Intravenous 5 doses: 5 mL of MenSCs-derived secretome diluted in 100 mL of normal saline	On days 1, 2, 3, 4 and 5	[39]
Chu et al.	UC	NTA, TEM, WB	Exosomes (30–200 nm)	Inhalation 2 doses: 7.66e + 0.8 to 7.00e + 0.7 particles/ml diluted in 5 ml with 0.9% sodium chloride	After 24 h of nebulization	[40]
Varkouhi et al.	UC	Flow cytometry, TEM, DLS	EVs (71.8–47.7 nm)	Intravenous 1 dose: 10 × 10^8^ EVs/kg	After 48 h of *E. coli* instillation	[41]
Cloer et al.	Size-exclusion chromatography	NTA, TEM, WB, Atomic force microscopy, Immunogold imaging, and phospholipid analysis	EVs (50–350 nm)	Intravenous 3 doses over 3 days: 250pmol/kg	After 3 h of infection	[42]
Park et al.	UC	NTA, Flow cytometry, SEM	MVs (180 nm)	Intravenous 1 dose: 200 – 400 µl of EVs	After 1 h of injury	[43]
Yu et al.	Total exosome isolation reagent (Invitrogen)	WB, TEM, NTA	Exosomes (30–150 nm)	Intravenously and Incubation 1–2 doses; 0, 25, 50, and 100 µg/ml of EVs	1 h before and immediately after mechanical ventilation	[44]
Mizuta et al.	ExoQuick-TC exosome precipitation kit	WB and DLS	Exosomes (30–120 nm)	Intravenous and coculture	30 min before histone injection and 24 h after seeding	[45]
Tang et al.	UC	TEM	MVs (200 nm)	Intratracheal or Coculture 1 dose: 30 µl	After 24 h of seeding or LPS administration	[46]
Wei et al.	Exosome isolation kit (Invitrogen)	TEM, WB	Exosomes (60 nm)	Intratracheal 1 dose: 50 µg of hucMSCs-exosomes or HFL-1-exosomes	After 4 h after LPS administration	[47]
Zhou et al.	Total exosome isolation reagent (Invitrogen)	NTA, WB	Exosomes (30–120 nm)	Intratracheal 1 dose: 70 µg of EVs	After 24–48 h of injury	[48]
Zhu et al.	UC	TEM	MVs (200 nm)	Intratracheal or Intravenous 1–2 doses: 30 or 60 µl of EVs	After 12 h of lung injury	[49]
Kaspi et al.	Tangential flow filtration (TFF) using the Krosflo KR2i system	NTA, MACSPlex exosomes kit using CytoFlex FACS, TEM	sEVs (146 and 114 nm)	Intratracheal 3 doses over 3 days: 50 µl (2.0 × 10^10^ vesicles/ml) Exo MSC or Exo MSC-NTF	After 3 h of LPS administration.	[50]
Deng et al.	UC	TEM, NTA, and WB	Exosomes (80–150 nm)	Intratracheal and intraperitoneal 1 dose: 50 or 100 µg exosomes in 10 µl PBS	After 1 h of LPS administration	[51]
Wang et al.	UC	NTA, DLS, TEM,	Exosomes (50–150 nm)	Intratracheal or tail vein 1 dose: MSC-EVs (100 µg/mL) or 50 µg	After 30 min of LPS administration	[52]
Chen et al.	UC	TEM, flow cytometry	MVs (200 nm)	Intratracheal 1 dose; 4 mg/kg	After 48 h or 1 week	[53]
Xu et al.	UC	TEM, NTA, WB	Exosomes (50–200 nm)	Intratracheal 1 dose; 50 µl in 1 mL EVs from 5 × 10^6^ cell	After exposure to phosgene	[54]
Monsel et al.	UC	WB, TEM	MVs (200 nm)	Intratracheal or Intravenous 3 doses: EVs administered intratracheal (30 or 60 µl) or intravenously (90 µl)	After 4 h of *E. coli* induction	[55]
Khatri et al.	UC	TEM, flow cytometry	EVs (100 nm)	Intratracheal or Incubation 1 dose: 10 µg/mL of EVs added to the culture medium, or 80 µg/kg of EVs, administered via intratracheal injection	After 12 h SwIV infection	[56]
Gao et al.	UC	WB, TEM, NTA	EVs (170 nm)	Intratracheal or Incubation 1 dose; 1 × 10^9^ EVs added to the culture medium, or 2.5 ~ 2.8 × 10^10^ EVs in 20 µL PBS	After 24 h of seeding and 1 h after PBS/PM2.5 exposure	[57]
Silva et al.	UC	NTA, TEM, WB	EVs (100–700 nm)	Tail vein 1 dose: 10 µL per 1 × 10^6^ cells	After 4 h of injury	[58]
Xu et al.	UC	TEM, WB	Exosomes (30–100 nm)	Tail vein 1 dose:100 µg of exosomes in 0.2 mL	Not specified	[59]
Liu et al.	ExoQuick exosome precipitation kit	NTA, WB, TEM	Exosomes (40–160 nm)	Tail vein 1 dose; 800 µg of EVs	After 24 or 48 h of burn	[60]
Huang et al.	UC	NTA, TEM, WB	EVs	Tail vein 1 dose: 100 µg/200 µl of EVs	After 30 min of LPS administration	[61]
Silva et al.	UC	SEM, DLS, NTA	EVs	Jugular vein 1 dose: EVs from 10^5^ cells	After 24 h after LPS administration	[62]
Shi et al.	UC and 12% PEG	TEM, NTA, WB	EVs (50–400 nm)	Inhalation 1 dose per group: 2.0 × 10^8^ particles (first cohort), 4.0 × 10^8^ particles (second cohort), 8.0 × 10^8^ particles (third cohort), 12.0 × 10^8^ particles (fourth cohort) or 16.0 × 10^8^ particles (fifth cohort).	After 2 h of *P. aeruginosa* instillation	[63]
Morrison et al.	UC	Flow cytometry	EVs (Less than 4 μm)	Intranasally 1 dose: 2.5 × 10^5^ AMs/mouse	After 4 h of lung injury	[64]
Li et al.	ExoEasy Maxi kit	TEM, WB	Exosomes (30–120 nm)	Incubation 1 dose: 300 µg/ml	After 24 h after seeding	[65]
Sui et al.	UC	TEM, WB, NTA	Exosomes (150 nm)	Incubation 1 dose: 20 µg	After 24 h of LPS administration	[66]
Park et al.	Size-exclusion chromatography	NTA, TEM, WB	EVs (121.8 nm)	Incubation 1 dose: 250 ng, 1 µg, and 1.75 µg of EVs	After 1 h of SARS-COV-2 infection	[67]
Kim et al.	UC	NTA, Tunable Resistive Pulse Sensing, SEM, TEM, AFM	Exosomes and EVs (20–100, 100–300 nm)	Incubation 1–2 doses; ranging from 6 × 10^5^–1.5 × 10^7^ particles per ml	After 24 h of seeding	[68]
Yi et al.	UC	NTA, TEM, Flow cytometry	Exosomes (30–120 nm)	Intravenous/coculture/Jugular vein 1 dose; 1 µg/100 µL or 100 µg/200 µL	After 24 h of seeding	[69]
Hu et al.	UC	Flow cytometry, SEM, NTA	MVs (185 nm)	Incubation 1–2 doses: 30 or 60 µl of EVs	After 24 h of seeding	[70]
Wu et al.	UC	NTA, WB, TEM	Exosomes (30–110 nm)	Intravenous/Tail vein/Intratracheal 1 dose: 100 µg diluted in 200 µl PBS	Not specified	[71]
Wang et al.	UC	Flow cytometry, TEM	MVs (200 nm)	Coculture 1 dose; unspecified concentration of EVs, added to the culture medium	After reaching confluency	[72]
Fang et al.	Anion exchange chromatography	NTA, WB, TEM, flow cytometry	sEVs (50–150 nm)	Intraperitoneal 1 dose: 1.5 × 10^10^ iPSC-MSC-sEV	On day 20 and 2 h before the challenge on day 21 and day 22	[73]
Potter et al.	UC	Flow cytometry, DLS	EVs (0.2, 0.5, 2.0, and 3.0 μm)	Not specified 30 µg of MSC EVs	After the shock period	[74]
Zhao et al.	UC	NTA, WB, TEM	EVs	Inhalation/tail vein 3 doses: 50 µg MSC-EVs diluted in 50 µL saline	After 3 h of LPS administration	[75]
Xia et al.	UC	NTA, WB, TEM	Exosomes (50–150 nm)	Tail vein 1 dose: 10 µg/mL AdMSC-Exos in 200 µL PBS	After 4 h of infection	[76]
Ikhlas et al.	UC	BCA	Exosomes/MVs	Incubation 1 dose: 3.5 µg for 200,000 cells	After 48 h of treatment	[77]
Liu et al.	Total exosome isolation reagent (Invitrogen)	NTA, TEM, WB	Exosomes (108 nm)	Intravenous/Intratracheal 1 dose: 50 µl exosomes	After 4 h of LPS administration	[78]
Mao et al.	UC and 30% sucrose/D2O cushion	BCA, NTA, TEM	Exosomes	Tail vein 1 dose: 20 mg/kg diluted in 200 µL PBS	After 24 and 72 h of SM administration	[79]
Chen et al.	UC	TEM, Flow cytometry	MVs	Intratracheal 1 dose: 10 µL containing 1 × 10^6^	After 48 h of BLM administration	[80]
Ibrahim et al.	Ultrafiltration	NTA, SEM, WB	EVs	Incubation	After 48 h of treatment	[81]

*UC* Ultracentrifugation, *DLS* Dynamic light scattering, *TEM* Transmission Electron Microscopy, *NTA* Nanoparticle Tracking Analysis, *SP-IRIS* Single-Particle Interferometric Reflectance Imaging Sensor, *SEM* Scanning Electron Microscopy, *BCA* bicinchoninic acid assay, *LPS* Lipopolysaccharide

### Stem Cell-EV Intervention Characteristics

**Fig. 3:**
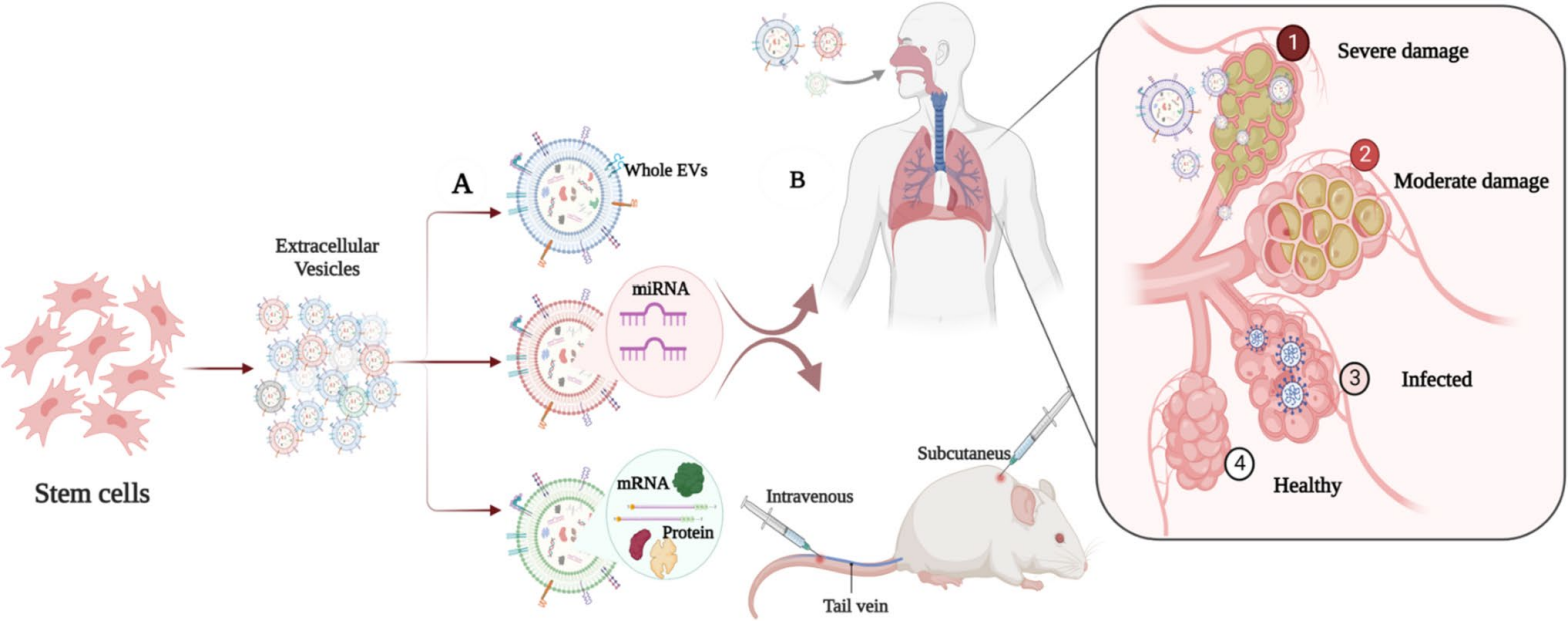
Stem cell EVs as therapeutics for COVID-19. **A** EVs derived from stem cells of various origins were investigated in the form of whole EVs or specific EV-associated cargos. **B** EVs are administered via different routes, in both clinical and preclinical studies

 Twenty-two studies out of the forty-eight selected studies used EV terminology based on the guidelines of the International Society for Extracellular Vesicles (ISEV) [82]. Table 1 shows the separation, characterization, reported nomenclature and size, dosage, and mode of administration of EVs, along with the time of assessment, for all studies. Separation methods included ultracentrifugation (the most common procedure, 66.7%), commercially available kits based on precipitation reagents (14.6%), size exclusion or anion exchange chromatography (6.3%), ultrafiltration (UF) (2.8%), combined methods including UC with UF (4.2%), UC with sucrose cushion (4.2%), and UC with PEG (2.8%) (Fig. 2C). Likewise, as recommended by ISEV, various characterization methods were involved, including transmission (75%) or scanning electron microscopy (10.4%), particle tracking analysis (66.7%), western blotting (52.1%), flow cytometry (35.4%), dynamic light scattering (10.4%), resistive pulse sensing (4.2%), and atom force microscopy (4.2%) (Fig. 2D). It is noteworthy that all the included studies reported the methods used in EV characterization, while only four studies used a single characterization procedure [39, 46, 49, 77]. Regarding the route of EV administration, EVs are most commonly administered to humans by inhalation or intravenous administration, while injection into the tail or jugular veins or intratracheal or intraperitoneal administration is also used in animal models (Fig. 2E). EVs were administered to humans one to five times but in animal models in just one or two doses. Throughout this review, EV therapy was in the form of whole EVs or specific EV-derived molecular cargos (i.e., miRNA, mRNA, or protein) that were isolated from EVs and tested and/or evaluated for their potential antiviral and therapeutic effects (Fig. 3).

### Stem Cell EVs as COVID-19 Therapeutics

**Fig. 4:**
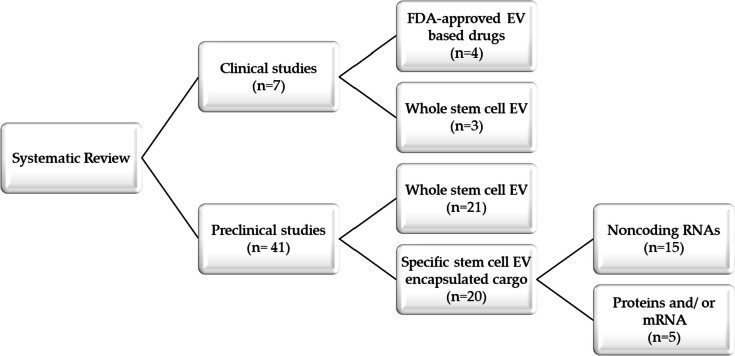
Illustration of the included studies classification. Throughout the systematic review, the included studies are classified into clinical (n = 7) and preclinical (n = 41) studies. Each group was subclassified based on the type of molecule used in the intervention. In clinical trials: whole stem cell EVs (n = 3) and EV-based drugs (n = 4) were used, while in preclinical studies: whole stem cell EVs (n = 21) or specific EV encapsulated noncoding RNA (n = 15) or encapsulated proteins or RNA (n = 5) were tested

 The included studies were classified into seven clinical and forty-one preclinical studies (Fig. 4). The seven studies evaluated the safety and/or efficiency of EVs against SARS-COV-2 in acute or long-hauler patients. Among them, four studies targeted COVID-19 mild, moderate, severe, or long-hauler patients using FDA-approved EV-based drugs (Zofin and Exoflo) derived from human amniotic fluid or bone marrow MSCs [35–38]. Similarly, three other studies highlighted the feasibility, tolerance, and safety of human umbilical, menstrual, and adipose MSC-derived EVs in alleviating SARS-CoV-2 [34, 39, 40]. Table 2 shows patient characteristics, EV source, effects, and outcomes. In these studies, EV-based drugs were shown to have no adverse events with improved oxygen saturation level, survival rates, SOFA and Glasgow scores, partial pressure of arterial oxygen to fraction of inspired oxygen (PaO2/FiO2), and absolute lymphocyte count (ALC). Moreover, they also improved immunocompetence by reducing neutrophil infiltration as well as the pro-inflammatory and anti-inflammatory cytokine storm, including tumor necrosis factor-alpha (TNF-α), interleukin-6 (IL-6), d-dimer, platelets, and c-reactive protein (CRP) [34–39].

**Table 2:** Characteristics of studies using stem cell EV-based therapeutics as an anti-COVID19 intervention

Study (Year - country)	Patients’ characteristics	Model (Induction)	Source of EVs	EV effects	EV treatment outcome summary	Reference
Zhu et al. (2022- USA)	Severe (n = 7)	SARS-CoV-2	Human adipose MSC	↑ Lymphocyte counts ↓CRP, IL-6, LDH Normal ALT and creatinine	haMSC‑EV inhalation is feasible and well-tolerated in COVID‑19 patients, with no evidence of prespecified adverse events, immediate clinical instability, or dose‑relevant toxicity.	[34]
Mitrani et al. (2021-USA)	Severe (n = 3)	SARS-CoV-2	Human amniotic fluid	↑ Oxygen saturation ↓ TNF-α, IL-6, D-Dimer, and CRP. Improved SOFA score, PaO2/FiO2, Glasgow score, and creatinine levels	Human amniotic fluid-derived nanoparticles as a safe and potentially efficacious treatment for respiratory failure induced by COVID-19 infection	[35]
Mitrani et al. (2021-USA)	Long hauler (n = 1)	SARS-CoV-2	Human amniotic fluid	↑ Oxygen saturation and monocyte ↓TNF-α, IL-6, D-Dimer, platelets, and CRP	Zofin acts as a potentially safe and therapeutically efficient treatment option for the growing number of COVID-19 long-hauler patients	[36]
Bellio et al. (2021-USA)	Mild to moderate acute (n = 8)	SARS-CoV-2	Human amniotic fluid	↑ALC ↓CRP, IL-6, TNF-α, cough, shortness of breath No abnormalities in CBC, CMP, and d-dimer	Zofin is a feasible, safe, and potentially efficacious therapy for patients with mild-to-moderate COVID-19 who are at increased risk for progression, including the need for hospitalization, ventilation, and death	[37]
Sengupta et al. (2020- USA)	Severe (n = 27)	SARS-CoV-2	Human bone marrow MSCs	↑ Absolute lymphocyte count, PaO2/FiO2 ratio, and survival rate ↓ CRP, ferritin, and D-dimer, absolute neutrophil count. Improved SOFA score, Glasgow score, and creatinine levels	ExoFlo is a promising therapeutic safe candidate for severe COVID-19 due to its capacity to restore oxygenation, downregulate cytokine storm, and reconstitute immunity	[38]
FKazerooni et al. (2022- Iran)	Severe (n = 30)	SARS-CoV-2	Human MenSCs	↑ Oxygen levels, survival rate, lymphocytes count ↓CRP, LDH l, D-Dimer, and ferritin	MenSC-derived secretome is a safe and feasible therapeutic strategy severe COVID19 patients	[39]
Chu et al. (2022- China)	COVID-19 pneumonia (n = 7)	SARS-CoV-2	Human umbilical cord MSCs	↓CRP and NK cells ↑IFN-γ, IL-17 A and TH19. No allergic reactions or adverse events. No change in oxygen saturation level, total white blood cell count, lymphocyte count, fever, or shortness of breath	Nebulization of MSC-derived EVs at early stages of COVID-19 is a simple, safe, and effective treatment for patients	[40]

*MSC* Mesenchymal stem cells, *ADCS* Adipose-derived stem cell, *MenSC* Menstrual stromal cells, *sEVs* Extracellular vesicles, *TNF-α* Tumor necrosis factor-alpha, *IL-6* Interleukin 6, *CRP* C-reactive protein, *PaO2/FiO2* partial pressure of arterial oxygen to fraction of inspired oxygen ratio, *CBC* complete blood count, *CMP* complete metabolic panel, *ALC* absolute lymphocyte count, *LDH* lactate dehydrogenase, *IFN-γ* Interferon gamma, *TH19* T helper 19

### Stem Cell EVs in ARDS, ALI, and Pneumonia Models

Twenty-one studies out of 41 preclinical studies used whole MSC EVs to treat model systems without identifying specific EV-based molecules responsible for any observed effects (Fig. 4) [41–44, 50, 51, 54–57, 62–65, 68, 73–76, 83]. As shown in Table 3, in all studies except one, ARDS or ALI models were induced by a variety of inflammation inducers in perfused lungs. Overall, EV administration improved survival rates and cellular repair, albeit not significantly reducing lung injury scores, as indicated by lessened inflammation, alveolar congestion, and cell permeability damage [41–44, 51, 54–57, 68, 76]. Ameliorated inflammation was evaluated by measuring neutrophil infiltration, M2 macrophage polarization, apoptotic macrophages [64, 73, 76], and proinflammatory and/or anti-inflammatory cytokines [50, 63, 65, 75, 76]. As recapped in Table 3, EVs improved mitochondrial respiration and ATP turnover [58, 76] and upregulated anti-inflammatory interleukin (IL-10), arginase-1 (Arg-1), keratinocyte growth factor (KGF), and prostaglandin E2 (PGE2) [54–56, 62, 63, 75, 76]. Adipose MSC EV preparations alleviated ALI and improved tissue integrity and pathological scores through mitochondrial DNA (mDNA) transfer [58, 76]. Additionally, EV treatment restored endothelial cell‒cell adhesion by increasing the levels of the adherens junction proteins VE-cadherin and ß-catenin [44, 74]. They also improved various respiratory functions, such as tidal volume (TV), peak inspiratory flow (PIF), peak expiratory flow (PEF), and 50% forced expiratory flow (EF50) [54]. In contrast, EV treatment significantly downregulated many proinflammatory cytokines, including IL-1β [50, 51, 62, 75, 76], IL-6, IL-8, IL-4, IL-5, IL-13, macrophage chemoattractant protein-1 (MCP-1), and RANTES [50, 73, 75, 76]. Similarly, inflammatory mediators such as tumor necrosis factor-α (TNF-α), macrophage inflammatory protein 2 (MIP-2), nuclear factor kappa B subunit 1 (NF-kB), and keratinocyte-derived chemokines were reported to be significantly downregulated [41–44, 50, 51, 54–58, 62–65, 68, 75, 76]. Modest expression of proteins in bronchoalveolar lavage fluid (BALF) was also reported. These included coagulation mediators such as tissue factor (TF), thrombin–antithrombin complex (TAT) [50], alveolar epithelial injury indicators (i.e., receptors for advanced glycation end products (RAGE)) [58], and lipid peroxidation measures (i.e., 4-hydroxynonenal (4-HNE)) [42]. EVs were reported to alleviate lung edema and hemorrhage as measured by decreased matrix metalloproteinase (MMP)-9 expression levels and lung tissue wet-to-dry ratio [44, 51, 54]. EVs also have a dramatic effect on glycolysis-related proteins such as hypoxia-inducible factor 1 (HIF-1a), hexokinase 2 (HK2), pyruvate kinase isoform M2 (PKM2), glucose transporter 1 (GLUT1), lactic acid, ATP, and lactate dehydrogenase A (LDHA) [51]. Interestingly, EVs preserved the lung structure and the alveolar-capillary barrier by reducing early apoptosis and necrosis, as indicated by lower levels of reactive oxygen species (ROS) [57, 75, 76], nitric oxide [68], and inducible nitric oxide synthase (iNOS) [55, 62, 76]. Additionally, EVs maintain lung integrity by modulating the crosstalk between inflammation and oxidation in ALI by regulating major oxidative stress mediators, such as nuclear factor erythroid 2-related factor 2 (Nrf2), Toll-like receptor 4 (Tlr4), Hmox heme oxygenase-1 (HO-1), and Arg-1 [75, 76].

**Table 3:** Characteristics of studies into the role of stem cell EVs in interventions (no specific molecular mechanisms)

Study (Year - country)	Species	Model (Induction)	Source of EVs	EV effects	EV treatment outcome summary	Reference
Varkouhi et al. (2019-Canada)	SD rats	ALI (*Escherichia* *coli* –induced)	Human UC-MSC	↑ Survival, alveolar permeability, mononuclear phagocytes, and bacterial phagocytosis ↓ Alveolar-arterial oxygen gradient, protein concentrations, TNF-α, lung injury	MSC-derived EVs enhanced the capacity to attenuate E. coli–induced lung injury via enhancement of macrophage phagocytosis and macrophage killing of E. coli	[41]
Cloer et al. (2021-China)	Sprague‒Dawley (SD) rats	ALI (LPS-induced)	Human BM-MSCs	↓ IL-6, IL-10,4-HNE, LDH, and TNFα ↑ Histological appearance, alveolar space, ↓ Lung injury score, cell permeability, and damage and infiltrating macrophages, lymphocytes, and neutrophils. .	MSC-EVs reduce local and systemic inflammation and lung injury.	[42]
Park et al. (2019-Korea)	Ex vivo perfused human lung	ALI (*Escherichia coli* –induced)	Human BM-MSCs	↓TNFα, AFC, cellularity and blood, edema, interstitial thickening, neutrophil, CFU counts, and lung protein permeability	MSC EVs act as therapeutic in a clinically relevant human model of severe *E. coli* pneumonia in lungs with baseline injury.	[43]
Yu et al. (2020-China)	SPF C57BL/6 mice	ALI (Mechanical ventilation-induced)	Mice adipose-MSC	↑VE-cadherin, ß -catenin ↓TRPV4, IL-6, TNF-α lung injury score, W/D ratio, MPO activity, intracellular calcium ion concentration, microvascular hyperpermeability, and protein concentration in BALF	Adipose-derived EVs alleviate the pulmonary endothelial barrier injury and inflammatory response by inhibiting the TRPV4/Ca2 pathway.	[44]
Kaspi et al. (2021- Israel)	BALB/c mice	ARDS/ALI (LPS-induced)	Human BM-MSCs	↓ IFNγ, TNFα, IL-6 and RANTES. ↓ TF and TAT, wall thickness and fibrin accumulation, physical damage and neutrophil accumulation	EV MSC-NTF significantly improved lung function and pathology and rebalanced the immune response in the ARDS model	[50]
Deng et al. (2020-China)	C57BL/6 mice	ALI (LPS-induced)	Mice BM-MSCs	↓ IL-1b, IL-6, TNF- α, HIF- 1a, HK2, PKM2, GLUT1, lactic acid, ATP, and LDHA. ↓ Lung injury, wet/dry ratio, PaO2/FiO2 ratio, macrophages, and total BALF protein	BMSCs-derived EVs inhibited M1 polarization and promoted M2 polarization by inhibiting cellular glycolysis via inhibition of HIF-1a.	[51]
Xu et al. (2019-China)	SD rats	ALI (Phosgene-induced)	Mice BM-MSCs	↓TNF-a, IL-1b, IL-6, EEP, RI, and MMP-9 ↓ Total protein content, W/D ratio, hemorrhage, and edema ↑IL-10, TV, PIF, PEF, EF50, SP-C, and alveolar structure	MSC-derived EVs exerted the therapeutic effects on phosgene-induced ALI through inhibiting MMP-9 synthesis and upregulating SP-C	[54]
Monsel et al. (2015-USA)	C57BL/6 mice	ALI (*E. coli* Pneumonia–induced)	Human BM-MSCs	↓ WBC, neutrophils, total protein, MIP-2, histological score, bacterial load, lung homogenate, TNF-a, and nitric oxide synthase ↑ Survival, KGF, PGE2, transglutaminase 2 and IL-10	MVs derived from human MSCs were as effective as the parent stem cells in severe bacterial pneumonia.	[55]
Khatri et al. (2018-USA)	Commercial pigs	ALI (Influenza-virus induced)	Pig BM-MSCs	↓TNFα, influenza replication, apoptosis, lung injury score, total protein, and inflammatory cells infiltration ↑ IL-10	MSC-EVs possess anti-influenza and anti-inflammatory properties and attenuated influenza virus-induced ALI in a pig model	[56]
Gao et al. (2020-China)	SD rats	ALI (PM2.5 -induced)	Human adipose-MSC	↓ Apoptosis or necrosis, alveolar congestion, hemorrhage, edema, and alveolar destruction ↓ ROS and TNF- α in BALF	ADSCs-EVs could serve as efficient antioxidative and anti-inflammatory interventions and further protect rats from lung injury	[57]
Silva et al. (2021-USA)	C57BL/6 mice	ARDS/ALI (LPS-induced)	Human BM-MSCs	↓ IL-8, TNF-α, keratinocyte-derived chemokine, and RAGE ↑ Mitochondrial respiration and ATP production ↓ BALF total protein, total and differential cell counts, and neutrophils.	MSC-EVs attenuate lung injury and restore lung tissue mitochondrial respiration in the mouse ARDS model	[58]
Silva et al. (2019-Brazil)	C57BL/6 mice	ARDS/ALI (*Escherichia coli* LPS–induced)	Mice BM-MSCs	↑ Arginase and IL-10 ↓ Total cells, macrophages, alveolar collapse and neutrophil, edema, and collagen fiber content ↓ TNF-α, IL-6, iNOS, IL-1β, KC, VEGF, and TGF-β	MSC-EVs and MSC had different effects based on ARDS etiology, however, MSCs yielded greater overall improvement in ARDS in comparison to EVs derived from the same number of cells	[62]
Shi et al. (2021-China)	C57BL/6 and BALB/c mice	ALI (Pseudomonas aeruginosa-induced)	Human adipose-MSC	↑ Survival rate and IL-10 ↓ Influx of BALF, WBCs, neutrophils ↓ IL-6, TNF-α, and histological severity	haMSC-EVs were safe and exerted protective effects in severe pneumonia.	[63]
Morrison et al. (2017- UK)	C57BL/6 male mice	ARDS/ALI (LPS or BALF-induced)	Human BM-MSCs	↓ Total cell counts, neutrophil, TNF-α, and protein ↑ Macrophage oxidative phosphorylation	MSCs-EVs promote an anti-inflammatory and highly phagocytic macrophage phenotype through mitochondrial transfer.	[64]
Li et al. (2020-China)	SPF grade C57BL/6 mice	ALI (LPS-induced)	Mice BM-MSCs	↓TNF- α, IL-6, IL-10, Ho-1, GPX-1, NRF-2, NF-kB and GR	MSC-EVs could reverse ALI through the Nrf-2/ARE and NF-κB signaling pathways	[65]
Kim et al. (2019-Australia)	Cell lines (CMSC29 and DMSC23)	ALI (LPS-induced)	Human placenta-MSCs	↓IL-6, TNF-α, nitric oxide, and lung injury ↑ Migration and cellular repair	EVs were beneficial in promoting migration and reducing oxidative stress and inflammation.	[68]
Fang et al. (2020-China)	BALB/c mice	Allergic airway inflammation (Ovalbumin (OVA)-induced)	Human iPSC-MSCs	↓ IL-4, IL-5, and IL-13 and Mo-AMs ↓ Total inflammatory cells and eosinophils ↑ Apoptotic macrophages	MSC-sEV act as an alternative therapy for allergic airway inflammation by ameliorating Th2-dominant allergic airway inflammation through immunoregulation on pulmonary macrophages	[73]
Potter et al. (2018-USA)	C57BL6 mice	ALI (HS and Laparotomy- induced)	Human BM-MSCs	↓ Pulmonary vascular permeability, VE-cadherin junctions, and RhoA GTPase activity ↑ Actin stress fibers	MSC-EVs may potentially be used as a novel “stem cell-free” therapeutic to treat HS-induced lung injury	[74]
Zhao et al. (2022-China)	C57BL6 mice	ALI (LPS-induced)	Human UC-MSC	↓ IL-1α, IL-1β, IL-12, TNFα, MCP-1, iNOS, TLR4, NF-κB p65, Keap1, pathological scores and oxidative stress ↑ IL-10, Arg-1, Nrf2, HO-1and M2 macrophage polarization	MSC-EVs administration may attenuate COVID-19 by regulating inflammatory and oxidative mediators during macrophage activation and ALI.	[75]
Xia et al. (2022-China)	C57BL6 mice	ARDS/ALI (LPS-induced)	Human adipose-MSC	↓ Apoptosis, cell counts, neutrophils and monocyte-derived macrophages, protein leakage, MPO activity IL-6, IL-1β, TNF-α, iNOS, MHC II and mROS ↑ Proliferation, survival rate, OCR, IL-10, Arg-1, mtDNA, MMP, NDUFV2, OXPHOS activity, and ATP generation	AdMSC-EVs may alleviate ALI severity through mitochondrial DNA transfer.	[76]
Mao et al. (2021-China)	ICR mice	ALI/ARDS (Sulfur mustard-induced)	Mice BM-MSCs	↓ Lung injury, edema, BALF, W/D, apoptosis, epithelial damage ↑ Survival rate, Bcl-2, barrier function repair of adherens and tight junction integrity, GPRC5A regulated by hippo/YAP pathway	BMSC-EV is a possible alternative approach to stem cell-based therapy as they have protective effects against ALI by promoting alveolar epithelial barrier repair.	[ 80 ]

*SD* Sprague‒Dawley, *ARDS* Acute respiratory distress syndrome, *ALI* Acute Lung Injury, *HS* Hemorrhagic Shock, *MSC* Mesenchymal stem cells, *ADCS* Adipose derived stem cell, *LPS* Lipopolysaccharide, *EVs* Extracellular vesicles, *MVs* Microvesicles, *IL-1β* Interlukin-1beta, *IL-6* Interleukin six, *TNF-α* Tumor necrosis factor alpha, *NF-κB* Nuclear factor kappa, *MMP-9* matrix metalloproteinase, *IFNγ* Interferon gamma, *TF* tissue factor, *TAT* thrombin–antithrombin, *RAGE* receptor for advanced glycation end products, *4-HNE* 4-hydroxynonenal, *LDH* Lactate dehydrogenase, *BALF* bronchoalveolar lavage fluid, *WBC* White blood cells, *VE-cadherin* vascular endothelial – cadherin, *Ho-1* hmox1 heme oxygenase 1, *GPX-1* glutathione peroxidase 1, *NRF-2* nuclear factor erythroid derived 2, like 2, *GR* glucocorticoid receptor, *iNOS* nitric oxide synthase, *HIF- 1a* Hypoxia-inducible factor 1, *HK2* hexokinase 2, *PKM2* pyruvate kinase isoform M2, *GLUT1* glucose transporter 1, *LDHA* lactate dehydrogenase A, *Mo-AMs* monocytes-derived macrophages, *ROS* reactive oxygen species, *WBC* White blood cells, *W/D ratio* wet to dry ratio, *TRPV4* transient receptor potential vanilloid 4, *MPO* myeloperoxidase, *MDA* malondialdehyde, *EEP* end expiratory pause, *RI* Lung resistance, *SP-C* surfactant protein-C, *TV* tidal volume, *PIF* peak inspiratory flow, *PEF* peak expiratory flow, *EF50* 50% forced expiratory flow, *KGF* keratinocyte growth factor, *PGE2* prostaglandin E2, *MIP-2* monocyte inflammatory protein-2, *PaO2/FiO2 ratio* partial pressure of arterial oxygen to fraction of inspired oxygen ratio, *MCP-1* macrophage chemoattractant protein-, *Keap1* Kelch-like ECH-associated protein 1, *MMP* mitochondrial membrane potential, *OXPHOS* mitochondrial oxidative phosphorylation, *NDUFV2* NADH: ubiquinone oxidoreductase core subunit V2, *OCR* oxygen consumption rates

### Stem Cell EV Encapsulated Cargos Against Acute Lung Injury

#### Regulatory Noncoding RNAs

Different types of nonregulatory RNAs, including miRNAs, long noncoding RNAs (lncRNAs), and PIWI–interacting RNAs (piRNAs), have been proposed to ameliorate SARS-CoV-2 complications such as ALI as shown in Table 4. piRNAs are often 24–32 nucleotides in length, compared with 21–24 nucleotides for miRNAs, and their biogenesis does not depend on the Dicer machinery [84]. These RNAs function, especially in the germ line, when complexed with the PIWI-subfamily argonaute proteins. PIWI-piRNAs were reported to be encapsulated into neural stem cell EVs to promote antiviral innate and adaptive immunity against SARS-CoV-2 [77]. Another type of noncoding RNA is miRNAs, small noncoding RNAs that exert posttranscriptional regulation by recognizing partially complementary sequences in target mRNAs and thus suppressing the production of proteins. Fifteen intervention studies investigated EV miRNAs as having roles in treating SARS-COV-2 or ARDS/ALI [45, 47, 48, 52, 59–61, 66, 67, 69, 71, 77, 78, 80, 81]. Various miRNAs were reported to mediate antiviral responses related to chemokines, cytokine–receptor interactions, TNF-α, NF-κB, Toll-like receptors, and the Jak-STAT signaling pathways. MSC-EV-associated miRNAs miR-92a-3p, miR-26a-5p, miR-23a-3p, miR-103a-3p, and miR-181a-5p were reported to efficiently regulate the inflammatory response in SARS-COV-2 by modulating the NF-κB signaling pathway and p65 translocation [67]. Significant attenuation of lung injury was attributed to various miRNAs, including miR-150, miR-181, miR-126, miR-377-3p, miR-27a-3p, miR-30b-3p, and miR-451, which regulate different signaling pathways [47, 48, 52, 59, 60, 66, 69, 71]. miR-150 in particular was reported to downregulate several MAPK pathway proteins, such as p-Erk, p-JNK, and p-p38, causing a reduction in various proinflammatory cytokines and of neutrophils [59]. Additionally, through the lncRNA-p21/miR-181/SIRT1 pathway, lncRNA-p21 suppressed apoptosis and lung tissue injury by sponging miR-181 and upregulating sirtuin 1 (SIRT1) [66]. miR-126 in EVs from MSCs and endothelial progenitor cells was implicated in reducing endothelial damage, lung hemorrhage, and edema while increasing the animal survival rate. This effect was mediated by regulating the PI3K/Akt signaling pathway and inhibiting the inflammatory alarmin high-mobility group protein (HMGB1) and vascular endothelial growth factor (VEGF) [45, 48, 71]. The Toll-like receptor 4 (TLR4)/NF-κB signaling pathway was also reportedly regulated by EV miR-451, which in turn was said to reduce the inflammation found in injured lungs [60]. However, the red blood cell-specificity of miR-451 should be considered when interpreting this report. Of note, macrophage polarization was reportedly promoted via EV miR-16-5p, miR-127-3p, and miR-125b-5p. This effect was due to suppression of the expression of M1 markers IL-12 and chemokine receptor (CCR-7), in addition to various cytokines, including TNF-α, IL-1β, IL-10 and IL-6 [61]. Other purportedly EV-associated miRNAs, such as miR-377-3p, miR-27a-3p, and miR-30b-3p, were reported to promote autophagy and phagocytic activity and inhibit apoptosis by suppressing inflammatory serum amyloid A3 (SAA3) expression, eventually leading to amelioration of the induced lung damage [47, 52, 69].

**Table 4:** Characteristics of studies into the role of stem cell EV noncoding RNA in interventions

Study (Year - country)	Species	Model (Induction)	Source of EVs	RNA of interest	EV effects	EV treatment outcome summary	Reference
Mizuta et al. (2020-Japan)	C57BL/6 mice	ALI (Histone-induced)	Human ADSC	miR-126	↑ Survival, miR-126, and Akt phosphorylation ↓ Lung hemorrhage, edema, endothelial damage and apoptosis, vascular hyperpermeability, and PI3K/Akt pathway	ADSC-EVs decreased histone-induced endothelial damage in vitro and in vivo models via the PI3K/Akt signaling pathway	[45]
Wei et al. (2020-China)	C57BL/6 mice	ALI (LPS-induced)	Human Umbilical cord (UC)-MSCs and Human fetal lung-1 cells	miR-377-3p	↓ Lung morphology and BALF concentration ↓ IL-1β, IL-6, IL-17, and MCP-1 ↑ Autophagy	miR-377-3p released by hucMSCs-EVs ameliorates LPS-induced acute lung injury by targeting RPTOR to induce autophagy	[47]
Zhou et al. (2019-USA)	CD-1 outbred mice	ALI (LPS-induced)	Endothelial progenitor cells	miRNA-126	↓ Cell number, protein concentration, cytokines/chemokines, MPO activity, lung injury score, and pulmonary edema ↑ miRNA-126-5p ↓ Alarmin HMGB1 and VEGFα.	EPC EVs restore injured alveolar epithelium and exerted a beneficial effect on LPS-induced via the transfer of miRNA-126	[48]
Wang et al. (2020-China)	C57BL/6 mice	ALI (LPS-induced)	Human adipose-MSC	miR-27a-3p	↓ Il-1β, NFKB1, iNOS and TNF-α ↑ YM-1 and CD206, phagocytic activity ↓ Lung permeability, number of total cells and neutrophils	MSC-EVs mitigate acute lung injury by transferring miR-27a-3p to alveolar macrophages.	[52]
Xu et al. (2021-China)	C57Bl/6J mice	ALI (LPS-induced)	Mice BM-MSCs	miR-150	↓TNF-α, IL-6, IL-1β, caspase-3, Bax, p-Erk, p-JNK, p-p38, total proteins, and the wet/dry pulmonary weight. ↓Total cells, neutrophils, and macrophages. ↓ Inflammation, permeability, and apoptosis	EV derived miR-150 attenuates LPS-induced ALI by modulating the microvascular endothelial cells and MAPK pathways.	[59]
Liu et al. (2019-China)	SD rats	ALI (Burn-induced)	Human UC-MSC	miR-451	↓ TNF-α, IL-1β, IL-6, TLR4 and p-P65 ↑ MDA, MPO, and SOD ↓ Burn injury and apoptosis	HUCMSC-EV-derived miR-451 improves ALI via the TLR4/NF-κB pathway.	[60]
Huang et al. (2019-China)	C57BL/6 mice	ALI (LPS-induced)	Mice BM-MSCs	miR-127-3p miR-125b-5p	↓ Protein, total cells, neutrophils, and total macrophages ↑ IL-10, Arg-1, Ym-1 and miR-223-5p ↓ IL-6, IL-1β, TNF-α, iNOS, miR-127-3p and miR-125b-5p	Aging and young MSC-EVs have differential effects in alleviating acute lung injury and macrophage polarization.	[61]
Sui et al. (2021-China)	C57BL/6 mice	ALI (LPS-induced)	Mice BM-MSCs	lncRNA-p21 miR-181	↓ miR-181, epithelial cell apoptosis, lung tissue injury, inflammatory cytokines ↑ SIRT1, proinflammatory cytokines	MSC-derived EVs may be a new therapeutic strategy for the treatment of SALI via the lncRNA-p21/miR-181/SIRT1 pathway	[66]
Park et al. (2021-Korea)	Cell lines (LL24, Beas-2B, BV2, and SK-N-BE (2) C)	SARS-CoV-2 (LPS-induced)	Placenta-derived MSC- or placenta	miR-92a-3p, miR-26a-5p, miR-23a-3p, miR-103a-3p, miR-181a-5p	↓ IL-1β and IL-6 ↓ TNF-α, NF-κB phospho-p65, miR-92a-3p, miR-26a-5p, miR-23a-3p, miR-103a-3p, and miR-181a-5p. Unchanged total p65 expression.	EVs significantly reduced inflammation stimulated by LPS.	[67]
Yi et al. (2019-China)	C57BL/6 mice	ALI (LPS-induced)	Mice BM-MSCs	miR-30b-3p	↑ KGF and proliferation ↓ Apoptosis, edema and thickening, W/D, neutrophilic granulocytes, and MPO activity ↓ SAA3, IL-1β, TNF-α, IL-10, and IL-6	EV-derived miR-30b-3p decreases the expression of SAA3 in recipient ACEs, which promotes proliferation and inhibits apoptosis thereby protecting against ALI	[69]
Wu et al. (2018-China)	SD rats	ALI (LPS-induced)	Endothelial progenitor cell	miR-126	↓ Edema, hemorrhage, the thickness of the alveolar wall, neutrophils infiltration and alveolar spaces, lung injury scores, WDR and total protein content, and MDA ↑ Permeability, proliferation, capillary tube formation, angiogenic capacity, migration, and PaO2 in arterial blood	EPC-derived EVs could mimic the beneficial effect of EPCs and can be an innovative drug delivery system by encapsulating exosomal miR-126.	[71]
Ikhlas et al. (2021-USA)	Cell lines: (hACE2-A549, HEK293T, BHK21)	SARS-CoV-2	Mice hypothalamic neural stem cells (htNSCs)	PIWIL2-piRNA	↓ Sg-E of SARS-CoV-2 and viral replication ↑ Antiviral effect through PIWIL2-piRNA system	PIWI-piRNA system is vital for EV from certain cell types to express antiviral innate and adaptive immunity against SARS-CoV-2	[77]
Liu et al. (2021-China)	SD rats	ALI/ARDS (LPS-induced)	Rat BM-MSCs	miR-384-5p	↓ Alveolar macrophage viability loss, apoptosis, autophagy flux of alveolar macrophages, Beclin-1, W/D, TNF-α, IL-1β, and IL-6 ↑ Survival rate, miR-384-5p, pulmonary vascular permeability, IL-10	EV-derived miR-384-5p is a potential treatment target for ALI/ARDS as it alleviates autophagy stress	[78]
Chen et al. (2020-China)	SD rats	ALI (Bleomycin-induced)	Human Wharton’s jelly MSCs	miR-100	↑ Autophagy, miR-100, ↓ IL-6, IL-8, and TNF-α, total protein content, total cell numbers, neutrophil counts, apoptosis, and inflammation	MSC-EVs enhance autophagy and ameliorate ALI partially via delivery of miR-100	[80]
Ibrahim et al. (2022-USA)	Cell lines: (ASTEX, TEV1, Calu-3)	SARS-CoV-2	Human neonatal skin fibroblasts	miR-16	↓IL-6, NFkB, virus replication, RAAS dysregulation, PI3K/mTOR ↑miR-16, cytoprotective and antiviral effects	Engineered EVs serve as a therapeutic candidate for COVID-19 through suppressing PI3K/mTOR pathway	[81]

*SD* Sprague‒Dawley, *ARDS* Acute respiratory distress syndrome, *ALI* Acute Lung Injury, *MSC* Mesenchymal stem cells, *ADCS* Adipose-derived stem cell, *LL24* Human lung fibroblast line, *Beas-2B* human bronchial epithelial cell line, *BV2* mouse microglial cell line, *SK-N-BE (2)C* human neuroblastoma cell line, *LPS* Lipopolysaccharide, *EVs* Extracellular vesicles, *MVs* Microvesicles, *lncRNA* long noncoding RNA, *IL-1β* Interleukin-1beta, *IL-6* Interleukin six, *TNF-α* Tumor necrosis factor-alpha, *NF-κB* Nuclear factor-kappa, *IFNγ* Interferon-gamma, *Arg-1* Arginase-1, *MCP-1* Monocyte chemoattractant protein-1, *BALF* bronchoalveolar lavage fluid, *WBC* White blood, *SIRT1* Sirtuin 1, *SOD *Superoxide dismutase, *GSH* glutathione, *FZD6* Frizzled class receptor 6, *iNOS* nitric oxide synthase, *WDR* wet to dry ratio, *MPO* myeloperoxidase, *MDA* malondialdehyde, *VEGFα* vascular endothelial growth factor, *HMGB1* high-mobility group protein, *TLR4* Toll-like Receptor 4, *Sg-E* Subgenomic E region, *ASTEX* Activated Specialized Tissue Effector EVs, *TEV1* EVs from immortalized CDC EVs

#### Coding mRNAs and Proteins

Several studies identified EV mRNAs or proteins as contributing to therapeutic effects. In five studies, EVs were reported to promote the healing of lung injuries via hepatocyte growth factor (HGF), angiopoietin-1 (Ang1), or keratinocyte growth factor (KGF) [46, 49, 53, 70, 72] (Table 5). HGF associated with EVs was reported to alleviate acute lung injury by reducing apoptosis, pro- and anti-inflammatory cytokines, neutrophil infiltration, and total protein content BALF [53]. Additionally, it was reported that MSC-EVs play a role in regulating endothelial permeability partly by HGF, as evidenced by elevated levels of lung integrity VE-cadherin and occludin proteins [72]. EV-mediated transfer of angiopoietin-1 mRNA to injured cells induced the secretion of anti-permeability factors and reduced white blood cells, total protein, and inflammatory TNF-α in BALF [46, 70]. Likewise, EV-associated KGF was reported to have protective effects as efficient as those of the parent MSCs, as indicated by reduced levels of TNF-α, neutrophils, protein, permeability, and extravascular lung water (EVLW), as well as elevated levels of MIP-2 and IL-10 [49].

**Table 5:** Characteristics of studies into the role of stem cell EV mRNAs and/or proteins in interventions

Study (Year - country)	Species	Model (Induction)	Source of EVs	Gene of interest	EV effects	EV treatment outcome summary	Reference	
Tang et al. (2017-China)	C57BL/6 mice	ALI (LPS-induced)	Human BM-MSCs	Ang-1	↓ Influx of WBCs, neutrophils, BAL albumin, total protein, histological injury, and TNF-a ↑ IL-10	EVs had therapeutic effects on ALI, and their immunomodulatory properties on macrophages were partly mediated through their content of Ang-1 mRNA	[46]	
Zhu et al. (2014-USA)	C57BL/6 mice	ALI (*E. coli* Endotoxin-Induced)	Human BM-MSCs and Human Lung fibroblast (HLF)	KGF	↓WBC, neutrophils, protein, permeability, EVLW, and TNF-α ↑ MIP-2, IL-10	MSC-EVs produced similar protective effects as MSCs themselves through the transfer of KGF mRNA.	[49]	
Chen et al. (2019-China)	SD rats	ALI (Bleomycin-induced)	Human Wharton’s Jelly MSCs	HGF	↓ TNF-α and IL-6, cell apoptosis, histological injury, total protein content, WBC, and neutrophils ↑ HGF, AT1α, and CD31 levels in BALF, proliferation rate	MSC-EVs administration could effectively alleviate BLM-induced ALI via HGF in rats	[53]	
Hu et al. (2018-USA)	Cell lines (NHLF, HLMVECs)	ALI (Cytomix, a mixture of human IL-1β, TNF‐α, and IFN‐γ)	Human BM- MSCs	Ang1	↑ Protein permeability, ZO-1, VE‐cadherin, Ang1 mRNA, and S1P kinase1	MSC EVs had a therapeutic effect associated with the transfer of Ang1 from the MV to the injured HLMVECs with subsequent secretion of the anti-permeability factor	[70]	
Wang et al. (2017-China)	Cell lines (Endothelial cells)	ALI (LPS-induced)	Mice BM-MSCs	HGF	↓ Permeability, apoptosis, IL-6, and IL-10 ↑ Proliferation, integrity, VE-cadherin, and occludin	MSC-EVs regulate anti-inflammatory and inflammatory balance partly by HGF	[72]	

*SD* Sprague‒Dawley, *ARDS* Acute respiratory distress syndrome, *ALI* Acute Lung Injury, *MSC* Mesenchymal stem cells, *ADCS* Adipose-derived stem cell, *LPS* Lipopolysaccharide, *EVs* Extracellular vesicles, *MVs* Microvesicles, *HLMVECs* human lung microvascular endothelial cells, *IL-1β* Interleukin-1beta, *IL-6* Interleukin six, *TNF-α* Tumor necrosis factor-alpha, *NF-κB* Nuclear factor-kappa, *IFN-γ* Interferon-gamma, *S1P* sphingosine 1 phosphate, *BALF* bronchoalveolar lavage fluid, *WBC* White blood cells, *HGF* Hepatocyte growth factor, *KGF* keratinocyte growth factor, *VE-cadherin* Vascular endothelial cadherin, *EVLW* extravascular lung water, *MIP-2* monocyte inflammatory protein-2

### Quality Assessment and Risk of Bias

**Fig. 5:**
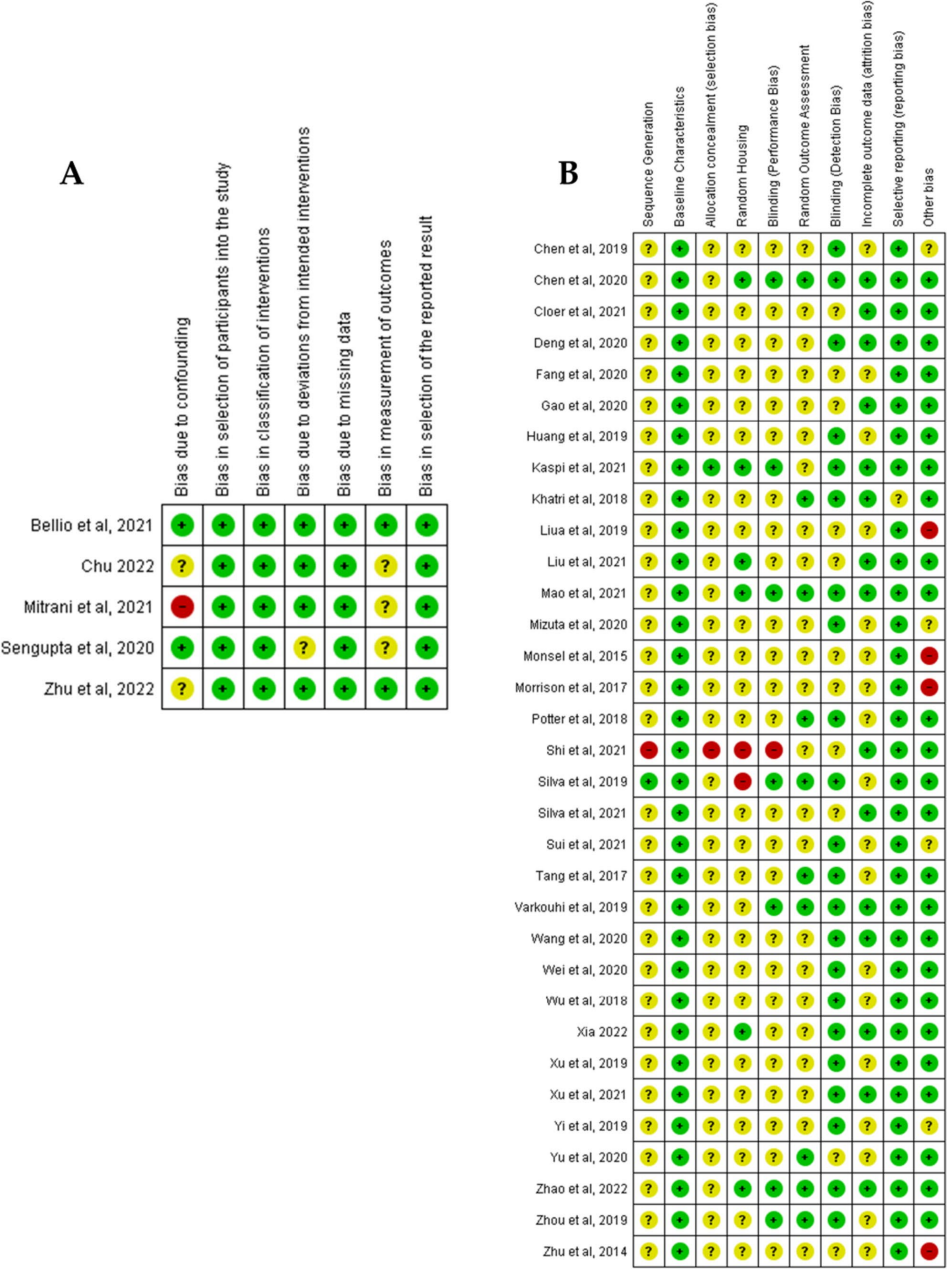
Risk of bias (RoB) assessment graph. **A** using the ROBINS-I RoB tool for cohort clinical studies, **B** using the SYRCLE’s RoB tool for preclinical studies

 Studies were subjected to quality assessment and categorized as having a “low”, “high”, or “unclear” risk of bias. As shown in Fig. 5A, human clinical studies scored low and moderate risk of bias using ROBINS-I due to measurement of outcomes and confounding biases. The in vivo animal studies assessed using SYRCLE had an unclear risk of bias, as most of them did not report details of sequence generation, allocation concealment, or random housing details. The in vitro studies assessed using modified SYRCLE for in vitro models showed a low risk of bias across all domains (Fig. 5B). The tables of the risk of bias assessment are provided in supplemental file 3. The CERQual tool that was used to rate the outcomes showed that the overall rating for assessment of confidence was “high” for inflammatory response and recovery of lung injury in the alveolar epithelium and lower in the other domains (Supplemental file 4). Finally, the PRISMA checklist was completed with further details for the review scoring (Supplemental file 5).

## Discussion

In this systematic review, we evaluate the reported role of stem cell EVs in targeting COVID-19 and its commonly induced complications, including ARDS, ALI, and pneumonia. Clinical studies showed that EVs derived from diverse stem cell sources could significantly ameliorate the clinical symptoms of lung injury induced by COVID-19 or complicating ARDS/ALI and reduce the time in the ICU or on mechanical ventilators. EV-treated patients had better survival rates, reversed hypoxia, and restored respiratory function and oxygenation index. Modulation of the cytokine storm was supported by downregulated proinflammatory cytokines, elevated anti-inflammatory cytokines, and decreased levels of immune cells, including neutrophils, lymphocytes, and macrophages.

### Stem Cell EVs are as Efficient as their Parental Cells Against COVID-19, ARDS, and Pulmonary Lung Injuries

MSCs were recently employed in 122 phase I and II clinical trials as a cell-based therapy against COVID-19, as detected by the Cochrane Central Register of Controlled Trials. In moderate to severe COVID-19 patients, administration of MSCs led to a significant increase in survival rates by reducing lung inflammation and modulating the immune system toward an anti-inflammatory status, with no serious complications reported [18, 85, 86]. In long-term follow-up, MSCs were shown to be safe and effective alternative therapeutic agents with a reliable recovery of lung lesions and COVID-19 symptoms. Minimal serious adverse effects during treatment or thereafter were reported [17]. Based on these data, MSCs were promoted to large-scale phase III clinical trials in subjects with varying severity profiles of COVID-19-induced ARDS and ALI to further evaluate their effect on mortality and long-term pulmonary disabilities [87]. Given the important role of EVs in MSC mechanisms of action, the outcome of MSC-EV-based clinical trials might also predict the efficacy of MSC-EV-based therapy against COVID-19, ARDS, pneumonia, or ALI. In the included studies, EVs were employed against COVID-19, ARDS, and/or ALI with or without identification of the roles of specific molecular cargo, such as miRNA, mRNA, or protein.

### Clinical Studies

In the included clinical studies, the whole EV cargo showed a significant capacity to maintain a reparative phenotype that restored lung vascular damage when used as a cell-free therapy against COVID-19, ARDS, pneumonia, and ALI. Sengupta et al. reported that administration of an MSC-derived EV preparation (Exoflo) in 24 patients diagnosed with severe COVID-19-induced ARDS restored their immunity and oxygenation capacity after the inflammation was ameliorated [38]. Similarly, in a clinical trial of 11 moderately to severely ill COVID-19 patients and a single long hauler, EVs derived from amniotic fluid (Zofin) were reported to be an accessible, feasible, safe, and efficacious treatment for respiratory failure induced by COVID-19 infection [35–38]. In another study by Zhu et al., aerosol inhalation of an EV preparation derived from human adipose‑derived MSCs suggested safety and efficiency in 7 severe COVID‑19 patients [34]. Likewise, Fathi-Kazerooni et al. showed that a menstrual stem cell-derived secretome was an efficient and feasible therapeutic that improved hypoxia, restored immune function and controlled the cytokine storm in 15 severe COVID-19 patients [39]. These findings were consistent with multiple systematic reviews that assessed the MSC therapeutic effect on severe COVID-19 patients [88–90]. This finding supports the efficacy of stem cell EVs as a cell-free therapy against different respiratory disorders, including COVID-19.

### Preclinical Studies

These findings were similar to those stated of experimental animal studies in which MSC-EVs were reported to significantly improve and mediate lung function and pathology via different pathways, including the TRPV4/Ca2, Nrf-2/ARE, and NF-κB signaling pathways [44, 65, 75]. Moreover, restoration of lung tissue function was mediated via mitochondrial transfer, resulting in reducing oxidative stress and promotion of an anti-inflammatory and highly phagocytic macrophage phenotype [64, 76]. Indeed, these findings aligned with similar mechanisms reported using stem cell-based therapy administration [91–94]. As described by Yan et al., MSC administration protected against ARDS and ALI at least in part by regulating Nrf2-Keap1-ARE signaling-mediated cell apoptosis [92]. Xiao et al. reported that MSCs reversed lung injury progression by blocking the activation of NF-κB pathways in ALI [93]. Additionally, Jackson et al. reported that mitochondrial transfer to alveolar macrophages was mediated via tunneling nanotubes (TNTs), leading to enhanced macrophage oxidative phosphorylation and phagocytosis [94]. Both MSCs and their EVs were found to be effective in diminishing inflammatory cytokines by inhibiting MMP-9 synthesis and upregulating SP-C [54]. MSC-EVs possess anti-inflammatory and antiviral properties that inhibit influenza virus-induced apoptosis and propagation in animal lung epithelial cells [56].

### EV RNAs and Proteins with COVID-19 Therapeutic Effects

MSC-EVs were shown to be superior, simpler, and clinically more convenient than their parental MSCs in COVID-19 therapy since EVs do not provoke immunological responses or lead toteratomas, and they protect their cargos against digestive circulating enzymes [95]. Moreover, whole MSC therapy might lead to significant vascular insufficiency, as cells may tend to aggregate intravascularly, and could synergize with COVID-19-induced vascular clots [96]. However, MSC-derived RNAs and proteins have been used as therapeutic targets in lung injuries, including COVID-19 [97, 98].

In comparison, in the analyzed preclinical studies, EV-miRNAs were reported to suppress endothelial damage, inflammatory interleukins, and apoptosis or to promote autophagy and macrophage polarization by mediating the lncRNA-p21/miR-181/SIRT1, PI3K/Akt, TLR4/NF-κB, and MAPK signaling pathways [45, 47, 48, 59, 60, 66]. Similar findings were reported by Li et al., who suggested that parental MSCs attenuated lung injury by a KGF-dependent PI3K/AKT/mTOR signaling pathway [99]. Similarly, MSCs mediate a therapeutic effects, in part, by many proteins, such as hepatocyte growth factor (HGF), angiopoietin-1 (Ang1), and keratinocyte growth factor (KGF) [100]. Perreau et al. and others reported that HGF expression levels could predict the severity of COVID-19 and that HGF could contribute to alleviating lung injury by suppressing the transforming growth factor-beta (TGF-β) signaling pathway [101–103]. While Adas et al. showed that KGF secreted by MSCs can reduce lung injury [103], similar protective effects were reported using MSC-MVs through the transfer of KGF mRNA to injured alveolar cells [49]. Lastly, Ang-1 mRNA transfer by MSC-EVs mediated the immunomodulatory properties of macrophages and was associated with a therapeutic effect on ALI [46, 70]. These findings were supported by a study by Lu et al., who reported that Ang-1-derived peptide inhibited apoptosis and improved endothelial cell survival, thus reducing inflammation induced by the SARS-CoV-2 virus [104].

### EV Characteristics and Application Considerations for Clinical Translation

Most of the EVs in the included studies, whether administered to patients, experimental animals, or in vitro, were reportedly smaller than 200 nm in diameter and derived mostly from MSCs. In at least partial agreement with the ISEV guidelines regarding EV characterization [105], forty-one studies in this review characterized EV preparation using three different protocols, [82]. It is worth mentioning that, currently, there are approximately 22 registered clinical trials of phases I, II, and III to evaluate the safety and efficiency of stem cell EVs against COVID-19. Only one systematic review has been published about in vivo animal studies of stem cell EVs against COVID-19, but it did not include any subjects infected by SARS-CoV-2 [106].

However, a specific effect of EVs has not necessarily been proven in these studies, which in general do not strictly establish EV preparation purity, integrity, efficacy, and specificity. This may be due to the various stem cells that were used as sources, separation through different procedures, non-standardized assessment and reporting of EV purity and integrity, and limited support for specific therapeutic effects of MSC-EVs versus non-MSC-EVs and/or co-separating non-EV factors.

Forty studies separated EVs from MSCs; however, these MSCs were derived from various sources, such as bone marrow, adipose tissue, and umbilical cord, possibly with an unclear safety profile. Forty-five out of 48 studies used either UC or precipitation reagents in EV separation. Both procedures are highly non-specific for EVs and were found to give significant variation in reported EV yield and size profile. In the clinical studies, although 4 studies reported safety and promising EV applications in severe COVID-19 cases, the sample sizes were small and in at least one case, commentators remarked on a lack of clarity about EV source and purity [107]. Similarly, in preclinical studies, the use of animals with different ages and unspecified breeding conditions may influence relevance of future human trials [108]. EVs might exert different actions in vitro and in vivo, and paracrine action of non-EV components of the MSC relesate might contribute to results [22]. Despite the recommendations of MISEV2018 [105], none of the studies established a biogenetic origin (e.g., of reported “exosomes” or “microvesicles” as opposed to a mixed EV population) or reported the presence of non-EV components or potential contaminants such as albumin, cytokines, or lipoprotiens from culture media or plasma/serum. Nevertheless, the unreported integrity and half-life of EVs before administration should be considered as an extra source of imposed variation.

EVs might also be engineered to have advantages over native EVs [81, 109, 110]. For example, cells might be engineered to produce EVs that modulate infection-related signaling pathways in recipient cells [81], or present targets for the SARS-CoV-2 spike protein and thus “sponge” the virus [109, 110]. These bioengineered EVs might enhance the overall yield, bioactivity, and half-life and improve the targeting effect for clinical applications; however, the extended half-life may induce adverse effects such as fibrosis. Thus, more future clinical studies should be carried out to test the validity, safety, and efficacy of these synthetic EVs.

EVs might also be engineered to have advantages over native EVs [81, 109, 110]. For example, cells might be engineered to produce EVs that modulate infection-related signaling pathways in recipient cells [81] or present targets for the SARS-CoV-2 Spike protein and thus “sponge” the virus [109, 110]. These bioengineered EVs might enhance the overall yield, bioactivity, and half-life, and improve the targeting effect for clinical applications, however, for instance, the extended half-life may induce adverse effects like fibrosis. Thus, more future clinical studies should be carried out to test the validity, safety, and efficacy of these synthetic EVs.

ISEV and the International Society for Cell and Gene Therapy (ISCT) urge that EV studies should consider multiple key points since EV research is relatively new and no adequate quality control and manufacturing obligations are yet in place [111]. Among these considerations are the source of stem cell EVs, optimal isolation technique, storage, dosing, and administration route [111]. Variations in these key points may affect the reproducibility of MSC-EVs in clinical research against COVID-19.

### Cell Source

The cell sources of EVs included human, animal, and in vitro studies were mostly either bone marrow or adipose MSCs. Although the mechanism by which EVs exert their antiviral or anti-inflammatory actions may differ depending on the source, all have shown promising efficacy against lung injuries, as illustrated earlier. However, it is worth mentioning that adipose-derived MSC-EVs were recently reported to increase the thrombosis risk more than bone marrow-derived MSC-EVs, potentially heightening the risk of microvascular injury syndrome in severe COVID-19 patients [112, 113]. Additionally, Huang et al. confirmed that EVs of different origins might have heterogeneous effects [61]. He showed that EVs derived from young MSCs had preferable effects in alleviating acute lung injury and macrophage polarization over EVs derived from aging MSCs in experimental animals [61].

### EV Separation Procedure

The procedure for EV separation or concentration could have a significant impact on the therapeutic outcome. Although different techniques, such as ultracentrifugation, size exclusion chromatography, precipitation, and immunoaffinity, were utilized in the included studies, ultracentrifugation was the most commonly reported. Furthermore, the ultracentrifugation sizing procedure and instrument varied from one study to another, which also led to different-sized EV populations. Each ultracentrifugation isolation procedure may hold advantages, such as high isolation efficiency, purity, and concentration, but they may also hold some disadvantages, such as isolating malfunctioning EVs. Harsh and rigorous purification procedures could even result in removal or damage of EV-intrinsic effectors or extrinsic factors that act with EVs to exert their functions [108]. Moreover, these different separation procedures may result in EVs of different sizes, concentrations, purity levels, and ultimately function. These differences could challenge reproducibility and complicate rapid EV clinical translation.

### EV Dosage and Administration

Major variation among studies may arise due to the absence of predefined patient enrollment criteria. Hence, patients involved in any study should be selected carefully by checking different clinical biomarkers, disease severity, age, and several other considerations. Another main contributor to variation is the dosage of stem cell EVs since it was equivalent to the dosage reported in stem cell-based therapy, among most studies, because the minimal amount of EVs required to induce therapeutic effects without being toxic is not yet defined. EV doses ranged from 1 up to 5 doses of varying concentrations diluted in either saline or sodium chloride. For intratracheal administration, the doses ranged from 1 up to 3 EV doses, while for inhalation and intravenous, they ranged from 1 up to 5 EV doses. Finally, the mode of EV administration is dependent on the disease’s severity, where IV is usually selected for direct and faster drug delivery into the bloodstream in severe COVID-19 cases. In contrast, oral route is often used in early COVID-19 patients when there is a need for effective and economical treatment that can be taken at home [114, 115]. However, the route of administration is also dependent on the drug properties, and the patient’s individual circumstances. Intravenous (IV) and inhalation routes were the most commonly used in humans [34–39, 116], while direct intratracheal application was common in preclinical studies. In COVID-19 patients, intravenous administration holds the potential to target not only injured lung cells, but also multiple organ failures induced by SARS-CoV-2, such as myocardial infarction and microvascular dysfunction [117, 118]. However, it is still debated to what extent IV administered EVs will reach injured lungs or other organs [119, 120]. EV inhalation, compared to IV, is simpler, less invasive, offers direct drug delivery to the lung, and can achieve higher drug concentrations at a lower overall dose. The intratracheal route delivers the treatment more directly.

### Evidence Profile

The included studies are methodologically sound, as evaluated by the SYRCLE risk of bias tool, which provided a very high rating for assessment of confidence in the domains most related to COVID-19, including inflammatory response and recovery of lung injury in the alveolar epithelium. This suggests the high likelihood of practical application of the findings from the selected animal studies to clinical settings. However, other domains of the SYRCLE risk of bias tool reported a lower level of confidence. This was potentially a result of unclear documentation of various parameters and methodological limitations such as blinding for assessors, group randomization, and concealment that ultimately impact the internal validity of the primary studies. Hence, there is a clear need for better documentation and rigorous methodological discipline in future studies in an effort to improve the reliability and credibility of the resultant publications.

### Limitations and Potential Solutions

The strength of our study is limited by the modest number of included COVID-19 patients and the high variation among the EV populations within the studies, which, in turn, limited the data available for a meta-analysis. However, available data on EV clinical trials support their application for effective COVID-19 therapy despite several challenges: (1) difficult and standardized EV separation procedures are lacking, with unclear implications for EV purity; (2) assessment of stem cell EVs in a clinical setting, such as their biodistribution, metabolism, excretion, etc., still cannot be performed; (3) there are no guidelines for large-scale EV production and quality control; and (4) optimal EV dose and dosing regimens must be determined. Further research is necessary to optimize EV production protocols, standardize dosage and administration routes, and conduct large-scale randomized controlled trials to definitively establish the efficacy and safety of EVs in a clinical setting. Additionally, exploring EVs derived from specific stem cell types tailored to target different stages and pathologies of COVID-19 holds promising potential for personalized medicine approaches.

## Conclusion

In conclusion, our systematic review of preclinical and clinical evidence demonstrates the immense potential therapeutic role of stem cell EVs in combatting COVID-19, particularly in mitigating the devastating complications of ARDS, ALI, and pneumonia. EVs derived from various stem cell sources hold a favorable safety profile and exhibit potential efficacy in combating COVID-19. Stem cell EVs induce anti-inflammatory properties in COVID-19 patients, evident in the suppression of the proinflammatory mediators, cytokine storm, and neutrophil infiltration, offer a critical approach to managing the detrimental inflammatory response associated with severe COVID-19. Beyond combating the acute effects of COVID-19, EVs display promise in facilitating patient recovery by promoting endothelial cell junction formation and reducing fibrin production, thereby mitigating pulmonary edema and improving lung function.

### Supplementary Information

Below is the link to the electronic supplementary material.

Supplementary Material 1 (docx 22.9 KB)

Supplementary Material 2 (docx 26.2 KB)

Supplementary Material 3 (docx 26.4 KB)

Supplementary Material 4 (docx 11.3 KB)

Supplementary Material 5 (docx 58.0 KB)

## Data Availability

All data presented in this review are totally available and present in the text.

## Abbreviations

*SARS-CoV-2*: Severe acute respiratory syndrome coronavirus 2
*ARDS*: Acute respiratory distress syndrome
*ALI*: Acute lung injury
*EVs*: Extracellular vesicles
*MVs*: Microvesicles
*ACE2*: Angiotensin-converting enzyme 2
*PRISMA*: Preferred Reporting Items for Systematic Reviews and Meta-analysis
*SYRCLE*: Systematic review center for laboratory animal experimentation
*CERQual*: Confidence in the Evidence from the Reviews of Qualitative research
*LPS*: Lipopolysaccharides
*UC*: Ultracentrifugation
*UF*: Ultrafiltration
*PEG*: Polyethylene glycol
*TEM*: Transmission electron microscopy
*SEM*: Scanning electron microscopy
*WB*: Western blot
*DLS*: Dynamic light scattering
*NTA*: Nano tracking analysis
*miRNA*: MicroRNA
*TNF-α*: Tumor necrosis factor-alpha
*IL-6*: Interlukin-6
*CRP*: C-reactive protein
*Arg-1*: Arginase-1
*KGF*: Keratinocyte growth factor
*PGE2*: Prostaglandin E2
*mDNA*: Mitochondrial DNA
*TV*: Tidal volume
*PIF*: Peak inspiratory flow
*PEF*: Peak expiratory flow
*EF50*: 50% forced expiratory flow
*MCP-1*: Macrophage chemoattractant protein 1
*NF-kB*: Nuclear factor kappa B
*BALF*: Bronchoalveolar lavage fluid
*TF*: Tissue factor
*TAT*: Thrombin–antithrombin complex
*RAGE*: Receptors for advanced glycation end products
*4-HNE*: 4-hydroxynonenal
*MMP*: Metalloproteinase
*HIF-1a*: Hypoxia-inducible factor 1
*HK2*: Hexokinase 2
*PKM2*: Pyruvate kinase isoform M2
*GLUT1*: Glucose transporter 1
*LDHA*: Lactate dehydrogenase A
*iNOS*: Inducible nitric oxide synthase
*Nrf2*: Nuclear factor erythroid 2-related factor 2
*Tlr4*: Toll-like receptor 4
*HO-1*: Hmox heme oxygenase 1
*lncRNAs*: Long noncoding RNAs
*piRNAs*: PIWI–interacting RNAs
*SIRT1*: Sirtuin 1
*HMGB1*: High-mobility group protein
*VEGF*: Vascular endothelial growth factor
*CCR-7*: Chemokine receptor
*SAA3*: Suppressing inflammatory serum amyloid A3
*HGF*: Hepatocyte growth factor
*Ang1*: Angiopoietin-1
*TNTs*: Tunneling nanotubes
*TGF-β*: Transforming growth factor-beta
*ISEV*: International Society for Extracellular Vesicles
*ISCT*: International Society for Cell and Gene Therapy
*IV*: Intravenous

## References

[1] Cucinotta, D., Vanelli, M. (2020). WHO declares COVID-19 a pandemic. *Acta bio-medica: Atenei Parmensis, 91*(1), 157–160. 10.23750/abm.v91i1.9397 PMC7569573 32191675

[2] Ranjan, K., Mohapatra, A. K. S., Venkataramana, K., Azam, M., Tiwari, R., & Dhama, K. (2022). Omicron (B.1.1.529 variant of SARS-CoV‐2); an emerging threat: Current global scenario. *Journal of Medical Virology - Wiley Online Library, 94*(5), 1780–1783. 10.1002/jmv.27561 PMC9015454 34964506

[3] Cascella, M., Rajnik, M., Aleem, A., Dulebohn, S. C., & Napoli, R. D. (2022). Features, evaluation, and treatment of coronavirus (COVID-19). StatPearls [Internet]. Treasure Island (FL): StatPearls Publishing. Available from: https://www.ncbi.nlm.nih.gov/books/NBK554776/32150360

[4] Hu B, Guo H, Zhou P, Shi ZL (2020). Characteristics of SARS-CoV-2 and COVID-19. Nature Reviews Microbiology.

[5] Zhou, F., Yu, T., Du, R., Fan, G., Liu, Y., & Liu, Z. (2020). Clinical course and risk factors for mortality of adult inpatients with COVID-19 in Wuhan, China: A retrospective cohort study. *The Lancet, 395*(10229), 1054–1062. 10.1016/S0140-6736(20)30566-3 PMC7270627 32171076

[6] Wu, C., Chen, X., Cai, Y., et al. (2020). Risk factors associated with acute respiratory distress syndrome and death in patients with Coronavirus Disease 2019 Pneumonia in Wuhan, China. *JAMA Internal Medicine, 180*(7), 934–943. 10.1001/jamainternmed.2020.0994 PMC7070509 32167524

[7] Ranieri, V., Rubenfeld, G. D., Thompson, B. T., Ferguson, N. D., et al. (2012). Acute respiratory distress syndrome: The Berlin definition. *Journal of the American Medical Association, 307*(23), 2526–2533. 10.1001/jama.2012.5669 22797452

[8] Spagnolo, P., Balestro, E., Aliberti, S., Cocconcelli, E., Biondini, D., Della Casa, G. (2020). Pulmonary fibrosis secondary to COVID-19: a call to arms? *The Lancet Respiratory Medicine, 8*(8), 750–752. 10.1016/S2213-2600(20)30222-8 PMC7228737 32422177

[9] Iacob, S., Iacob, D. G. (2020). SARS-CoV-2 treatment approaches: numerous options, no certainty for a versatile virus. *Frontiers in Pharmacology, 11*, 1224. 10.3389/fphar.2020.01224 PMC7479232 32982720

[10] Shouman, S., Zaher, A., Abdelhameed A., Elshaboury, S., Sakr, S., Fouda, B. E., et al. (2021). Cardiac progenitor cells. *Advances in Experimental Medicine and Biology, 1312*, 51–73. 10.1007/5584_2020_594 33159305

[11] Han, F., & Lu, P. (2020). Introduction for stem cell-based therapy for neurodegenerative diseases. *Advances in Experimental Medicine and Biology, 1266*, 1–8. 10.1007/978-981-15-4370-8_1 33105491

[12] Parekh, K. R., Nawroth, J., Pai, A., Busch, S. M., Senger, C. N., & Ryan, A. L. (2020). Stem cells and lung regeneration. *American journal of physiology. Cell physiology, 319*(4), C675–C693. 10.1152/ajpcell000362020 10.1152/ajpcell.00036.2020 PMC7654650 32783658

[13] El-Badawy, A., & El-Badri, N. (2015). Regulators of pluripotency and their implications in regenerative medicine. *Stem Cells and Cloning: Advances and Applications, 8*, 67–80. 10.2147/SCCAA.S80157 PMC4410894 25960670

[14] André Coelho, R. D. A., Branquinho, M. V., Guerreiro, S. G., Maurício, A. C. (2020). Mesenchymal stem cells (MSCs) as a potential therapeutic strategy in COVID-19 patients. Literature Research,* Cell and Developmental Biology, 8*, 1–13. 10.3389/fcell.2020.602647 PMC7710935 33330498

[15] Shi, L., Wang, L., Xu, R., Zhang, C., Xie, Y., Liu, K., et al. (2021). Mesenchymal stem cell therapy for severe COVID-19. *Signal Transduction and Targeted Therapy, 6*(1), 1–5. 10.1038/s41392-021-00754-6 PMC8424619 34497264

[16] Matthay, M., Calfee, C., Zhuo, H., Thompson, B., Wilson, J. G., Levitt, J., et al. (2019). Treatment with allogeneic mesenchymal stromal cells for moderate to severe acute respiratory distress syndrome (START study): A randomised phase 2a safety trial. *The Lancet, 7*(2), 154–162. 10.1016/S2213-2600(18)30418-1 PMC7597675 30455077

[17] Shi, L., Huang, H., Lu, X., Yan, X., Jiang, X., Xu, R., et al. (2021). Effect of human umbilical cord-derived mesenchymal stem cells on lung damage in severe COVID-19 patients: A randomized, double-blind, placebo-controlled phase 2 trial. *Signal Transduction and Targeted Therapy, 6*(1), 58. 10.1038/s41392-021-00488-5 PMC7873662 33568628

[18] Shi, L., Yuan, X., Yao, W., Wang, S., Zhang, C., Zhang, B., et al. (2022). Human mesenchymal stem cells treatment for severe COVID-19: 1-year follow-up results of a randomized, double-blind, placebo-controlled trial. *EBioMedicine, 75*(1-14), 103789. 10.1016/j.ebiom.2021.103789 PMC8709782 34963099

[19] Leng, Z., Zhu, R., Hou, W., Feng, Y., Yang, Y., Han, Q., et al. (2020). Transplantation of ACE2 - mesenchymal stem cells improves the outcome of patients with COVID-19 Pneumonia. *Aging and Disease, 11*(2), 216–228. 10.14336/AD.2020.0228 PMC7069465 32257537

[20] Ren, X., Wen, W., Fan, X., Hou, W., Su, B., Cai, P., et al. (2021). COVID-19 immune features revealed by a large-scale single-cell transcriptome atlas. *Cell, 184*(7), 1895–1913. 10.1016/j.cell.2021.01.053 PMC7857060 33657410

[21] Ahmed, S. H., Espinoza-Sánchez, N. A., El-Damen, A., Fahim, S. A., Badawy, M. A., Greve, B., et al. (2021). Small extracellular vesicle-encapsulated miR-181b-5p, mir-222-3p and let-7a-5p: Next generation plasma biopsy-based diagnostic biomarkers for Inflammatory Breast cancer. *PloS One, 16*(4), e0250642 10.1371/journal.pone.0250642 PMC8075236 33901254

[22] Witwer, K. W., Van Balkom, B. W., Bruno, S., Choo, A., Dominici, M., Gimona, M., et al. (2019). Defining mesenchymal stromal cell (MSC)-derived small extracellular vesicles for therapeutic applications. *Journal of Extracellular Vesicles, 8*(1), 1609206. 10.1080/20013078.2019.1609206 PMC6493293 31069028

[23] Laffey, J. G., & Matthay, M. A (2017). Fifty years of research in ARDS. Cell-based therapy for acute respiratory distress syndrome. Biology and potential therapeutic value. *American Journal of Respiratory and Critical care Medicine, 196*(3), 266–273. 10.1164/rccm.201701-0107CP PMC5549868 28306336

[24] Lener, T., Gimona, M., Aigner, L., Börger, V., Buzas, E., Camussi, G., et al. (2015). Applying extracellular vesicles based therapeutics in clinical trials - an ISEV position paper. *Journal of Extracellular Vesicles, 4*, 30087. 10.3402/jev.v4.30087 PMC4698466 26725829

[25] Ong, S. G., & Wu, J. C. (2015). Exosomes as potential alternatives to stem cell therapy in mediating cardiac regeneration. *Circulation Research, 117*(1), 7–9. 10.1161/CIRCRESAHA.115.306593 PMC4546704 26089361

[26] Dinh, P. U. C., Paudel, D., Brochu, H., Popowski, K. D., Gracieux, M. C., Cores, J., et al. (2020). Inhalation of lung spheroid cell secretome and exosomes promotes lung repair in pulmonary fibrosis. *Nature Communications, 11*(1), 1064. 10.1038/s41467-020-14344-7 PMC7048814 32111836

[27] Moher, D., Liberati, A., Tetzlaff, J., & Altman, D. G. (2009). Preferred reporting items for systematic reviews and meta-analyses: The PRISMA statement. *PLoS Medicine, 6*(7), e1000097. 10.1371/journal.pmed.1000097 PMC2707599 19621072

[28] Prospero [Internet]. National Institute for Health and Care Research (UK). 2011 Feb. Identifiers: CRD42022335053 and CRD42022336501. Stem cell-derived extracellular vesicles as anti-SARS-CoV-2 immunomodulatory therapeutics: a systematic review; 2022 May 30, Regenerative Capacities of Stem Cell-derived Extracellular vesicles Against COVID-19: A Systematic Review; 2022 July 5 [cited 2023 Jan]. Available from: https://www.crd.york.ac.uk/prospero/

[29] Salazar JW, Francisco S, McWilliams JM, Boston, Massachusetts A, Editor JIM, Wang TY, Department of Medicine UoC, Department of Health Care Policy HMS (2020). Setting expectations for clinical research during the COVID-19 pandemic. JAMA Internal Medicine.

[30] Sterne, J. A., Savović, J., Page, M. J., Elbers, R. G., Blencowe, N. S., Boutron, I., et al. (2019). RoB 2: A revised tool for assessing risk of bias in randomised trials. *BMJ (Clinical Research ed), 366*, l4898. 10.1136/bmj.l4898 31462531

[31] Sterne, J. A., Hernán, M. A., Reeves, B. C., Savović, J., Berkman, N. D., Viswanathan, M., et al. (2016). ROBINS-I: A tool for assessing risk of bias in non-randomised studies of interventions. *BMJ, 355*, 1–7. 10.1136/bmj.i4919 PMC5062054 27733354

[32] Hooijmans, C. R., Rovers, M. M., De Vries, R. B., Leenaars, M., Ritskes-Hoitinga, M., & Langendam, M. W. (2014). SYRCLE’s risk of bias tool for animal studies. *BMC Medical Research Methodology, 14*(43), 1–9. 10.1186/1471-2288-14-43 PMC4230647 24667063

[33] Lewin, S., Glenton, C., Munthe-Kaas, H., Carlsen, B., Colvin, C. J., Gülmezoglu, M., et al. (2015). Using qualitative evidence in decision making for health and social interventions: An approach to assess confidence in findings from qualitative evidence syntheses (GRADE-CERQual). *PLoS Medicine, 12*(10), e1001895, 1–18. 10.1371/journal.pmed.1001895 PMC4624425 26506244

[34] Zhu, Y. G., Shi, M. M., Monsel, A., Dai, C. X., Dong, X., Shen, H., et al. (2022). Nebulized exosomes derived from allogenic adipose tissue mesenchymal stromal cells in patients with severe COVID-19: A pilot study. *Stem cell Research & Therapy, 13*(1), 220, 1–10. 10.1186/s13287-022-02900-5 PMC9135389 35619189

[35] Mitrani, M. I., Bellio, M. A., Sagel, A., Saylor, M., Kapp, W., VanOsdol, K., et al. (2021). Case Report: Administration of amniotic fluid-derived nanoparticles in three severely Ill COVID-19 patients. *Frontiers in Medicine, 8*(583842), 1–8. 10.3389/fmed.2021.583842 PMC8010176 33816515

[36] Mitrani, M. I., Bellio, M. A., Meglin, A., Khan, A., Xu, X., Haskell, G., et al. (2021). Treatment of a COVID-19 long hauler with an amniotic fluid-derived extracellular vesicle biologic. *Respiratory Medicine case Reports, 34, *101502. 10.1016/j.rmcr.2021.101502 PMC8405236 34485048

[37] Bellio, M. A., Bennett, C., Arango, A., Khan, A., Xu, X., Barrera, C., et al. (2021). Proof-of-concept trial of an amniotic fluid-derived extracellular vesicle biologic for treating high risk patients with mild-to-moderate acute COVID-19 Infection. *Biomaterials and Biosystems, 4, *100031. 10.1016/j.bbiosy.2021.100031 PMC8611818 34841370

[38] Sengupta, V., Sengupta, S., Lazo, A., Woods, P., Nolan, A., & Bremer, N. (2020). Exosomes derived from bone marrow mesenchymal stem cells as treatment for severe COVID-19. *Stem Cells and Development, 29*(12), 747–754. 10.1089/scd.2020.0080 PMC7310206 32380908

[39] Fathi-Kazerooni, M., Fattah-Ghazi, S., Darzi, M., Makarem, J., Nasiri, R., Salahshour, F., et al. (2022). Safety and efficacy study of allogeneic human menstrual blood stromal cells secretome to treat severe COVID-19 patients: Clinical trial phase I & II. *Stem cell Research & Therapy, 13*(1), 96. 10.1186/s13287-022-02771-w PMC8899458 35255966

[40] Chu, M., Wang, H., Bian, L., Huang, J., Wu, D., Zhang, R., et al. (2022). Nebulization therapy with umbilical cord mesenchymal stem cell-derived exosomes for COVID-19 Pneumonia. *Stem cell Reviews and Reports, 18*(6), 2152–2163. 10.1007/s12015-022-10398-w PMC9166932 35665467

[41] Varkouhi, A. K., Jerkic, M., Ormesher, L., Gagnon, S., Goyal, S., Rabani, R., et al. (2019). Extracellular vesicles from Interferon-γ-primed human umbilical cord mesenchymal stromal cells reduce Escherichia coli-induced Acute Lung Injury in rats. *Anesthesiology, 130*(5), 778–790. 10.1097/ALN.0000000000002655 30870158

[42] Cloer, C., Roudsari, L., Rochelle, L., Petrie, T., Welch, M., Charest, J., et al. (2021). Mesenchymal stromal cell-derived extracellular vesicles reduce lung inflammation and damage in nonclinical acute lung injury: Implications for COVID-19. *PloS One, 16*(11), e0259732. 10.1371/journal.pone.0259732 PMC8592477 34780505

[43] Park, J., Kim, S., Lim, H., Liu, A., Hu, S., Lee, J., et al. (2019). Therapeutic effects of human mesenchymal stem cell microvesicles in an ex vivo perfused human lung injured with severe E. Coli Pneumonia. *Thorax, 74*(1), 43–50. 10.1136/thoraxjnl-2018-211576 PMC6295323 30076187

[44] Yu, Q., Wang, D., Wen, X., Tang, X., Qi, D., He, J., et al. (2020). Adipose-derived exosomes protect the pulmonary endothelial barrier in ventilator-induced lung injury by inhibiting the TRPV4/Ca 2 + signaling pathway. *American Journal of Physiology Lung Cellular and Molecular Physiology, 318*(4), L723–L741. 10.1152/ajplung.00255.2019 PMC7191475 32073873

[45] Mizuta, Y., Akahoshi, T., Guo, J., Zhang, S., Narahara, S., Kawano, T., et al. (2020). Exosomes from adipose tissue-derived mesenchymal stem cells ameliorate histone-induced acute lung injury by activating the PI3K/Akt pathway in endothelial cells. *Stem Cell Research & Therapy, 11*(1), 508. 10.1186/s13287-020-02015-9 PMC7691956 33246503

[46] Tang, X. D., Shi, L., Monsel, A., Li, X. Y., Zhu, H. L., Zhu, Y. G., et al. (2017). Mesenchymal stem cell microvesicles attenuate acute lung injury in mice partly mediated by Ang-1 mRNA. *Stem Cells, 35*(7). 10.1002/stem.2619 28376568

[47] Wei, X., Yi, X., Lv, H., Sui, X., Lu, P., Li, L., et al. (2020). MicroRNA-377-3p released by mesenchymal stem cell exosomes ameliorates lipopolysaccharide-induced acute lung injury by targeting RPTOR to induce autophagy. *Cell Death & Disease, 11*(9), 746. 10.1038/s41419-020-02976-y PMC7487066 32920597

[48] Zhou Y, Li P, Goodwin AJ, Cook JA, Halushka PV, Chang E (2019). Exosomes from endothelial progenitor cells improve outcomes of the lipopolysaccharide-induced acute lung injury. Critical Care.

[49] Zhu, Y. G., Feng, X. M., Abbott, J., Fang, X. H., Hao, Q., Monsel, A., et al. (2014). Human mesenchymal stem cell microvesicles for treatment of Escherichia coli endotoxin-induced acute lung injury in mice. *Stem Cells (Dayton Ohio), 32*(1), 116–25. 10.1002/stem.1504 PMC3947321 23939814

[50] Kaspi, H., Semo, J., Abramov, N., Dekel, C., Lindborg, S., Kern, R., et al. (2021). MSC-NTF (NurOwn®) exosomes: A novel therapeutic modality in the mouse LPS-induced ARDS model. *Stem Cell Research & Therapy, 12*(1), 72. 10.1186/s13287-021-02143-w PMC7814377 33468250

[51] Deng, H., Wu, L., Liu, M., Zhu, L., Chen, Y., Zhou, H., et al. (2020). Bone marrow mesenchymal stem cell-derived exosomes attenuate LPS-Induced ARDS by modulating macrophage polarization through inhibiting glycolysis in macrophages. *Shock, 54*(6), 828–843. 10.1097/SHK.0000000000001549 32433208

[52] Wang, J., Huang, R., Xu, Q., Zheng, G., Qiu, G., Ge, M., et al. (2020) Mesenchymal stem cell-derived extracellular vesicles alleviate Acute Lung Injury Via transfer of miR-27a-3p. *Critical care Medicine, 48*(7), e599–e610. 10.1097/CCM.0000000000004315 32317602

[53] Chen, W., Wang, S., Xiang, H., Liu, J., Zhang, Y., Zhou, S., et al. (2019). Microvesicles derived from human Wharton’s Jelly mesenchymal stem cells ameliorate acute lung injury partly mediated by hepatocyte growth factor. * International Journal of Biochemistry & cell Biology, 112*, 114–122. 10.1016/j.biocel.2019.05.010 31100425

[54] Xu, N., Shao, Y., Ye, K., Qu, Y., Memet, O., He, D., et al. (2019). Mesenchymal stem cell-derived exosomes attenuate phosgene-induced acute lung injury in rats. *Inhalation Toxicology, 31*(2), 52–60. 10.1080/08958378.2019.1597220 31068039

[55] Monsel, A., Zhu, Y. G., Gennai, S., Hao, Q., Hu, S., Rouby, J. J., et al. (2015). Therapeutic effects of Human mesenchymal stem cell-derived microvesicles in severe Pneumonia in mice. *American Journal of Respiratory and Critical care Medicine, 192*(3), 324–36. 10.1164/rccm.201410-1765OC PMC4584251 26067592

[56] Khatri M, Richardson LA, Meulia T (2018). Mesenchymal stem cell-derived extracellular vesicles attenuate Influenza virus-induced acute lung injury in a pig model. Stem Cell Research & Therapy.

[57] Gao, Y., Sun, J., Dong, C., Zhao, M., Hu, Y., Jin, F. (2020). Extracellular vesicles derived from adipose mesenchymal stem cells alleviate PM2.5-induced lung injury and pulmonary fibrosis. *Medical Science Monitor: International Medical Journal of Experimental and Clinical Research, 26*, e922782. 10.12659/MSM.922782 PMC7191958 32304204

[58] Silva, J. D., Su, Y., Calfee, C. S., Delucchi, K. L., Weiss, D., McAuley, D. F., et al. (2021). Mesenchymal stromal cell extracellular vesicles rescue mitochondrial dysfunction and improve barrier integrity in clinically relevant models of ARDS. *The European Respiratory Journal, 58*(1), 2002978. 10.1183/13993003.02978-2020 PMC8318599 33334945

[59] Xu, J., Xu, D., Yu, Z., Fu, Z., Lv, Z., Meng, L., & Zhao, X. (2021). Exosomal miR-150 partially attenuated acute lung injury by mediating microvascular endothelial cells and MAPK pathway. *Bioscience Reports, 42*(1), BSR20203363. 10.1042/BSR20203363 PMC8703023 34750610

[60] Liu, J. S., Du, J., Cheng, X., Zhang, X. Z., Li, Y., & Chen, X. L. (2019). Exosomal miR-451 from human umbilical cord mesenchymal stem cells attenuates burn-induced acute lung injury. *Journal of the Chinese Medical Association: JCMA, 82*(12), 895–901. 10.1097/JCMA.0000000000000189 PMC13047989 31800531

[61] Huang, R., Qin, C., Wang, J., Hu, Y., Zheng, G., Qiu, G., et al. (2019). Differential effects of extracellular vesicles from aging and young mesenchymal stem cells in acute lung injury. *Aging (Albany Ny), 11*(18), 7996–8014. 10.18632/aging.102314 PMC6781978 31575829

[62] Silva, J. D., de Castro, L. L., Braga, C. L., Oliveira, G. P., Trivelin, S. A., Barbosa-Junior, C. M. (2019). Mesenchymal stromal cells are more effective than their extracellular vesicles at reducing lung injury regardless of acute respiratory distress syndrome etiology. *Stem Cells International*, 8262849. 10.1155/2019/8262849 PMC6720722 31531026

[63] Shi, M. M., Yang, Q. Y., Monsel, A., Yan, J. Y., Dai, C. X., Zhao, J. Y., et al. (2021). Preclinical efficacy and clinical safety of clinical-grade nebulized allogenic adipose mesenchymal stromal cells-derived extracellular vesicles. *Journal of Extracellular Vesicles, 10*(10), e12134. 10.1002/jev2.12134 PMC8363910 34429860

[64] Morrison, T. J., Jackson, M. V., Cunningham, E. K., Kissenpfennig, A., McAuley, D. F., O’Kane, C. M. (2017). Mesenchymal stromal cells modulate macrophages in clinically relevant lung injury models by extracellular vesicle mitochondrial transfer. *American Journal of Respiratory and Critical care Medicine, 196*(10), 1275–1286. 10.1164/rccm.201701-0170OC PMC5694830 28598224

[65] Li, J., S, Deng, X., Ji, X., Shi, X., Ying, Z., Shen, K., Xu, D., Cheng, Z. (2020). Mesenchymal stem cell exosomes reverse acute lung injury through Nrf-2/ARE and NF-κB signaling pathways. *PeerJ, 8*, e9928. 10.7717/peerj.9928 PMC7505076 32999767

[66] Sui, X., Liu, W., Liu, Z. (2021). Exosomal lncRNA-p21 derived from mesenchymal stem cells protects epithelial cells during LPS-induced acute lung injury by sponging miR-181. *Acta Biochimica Et Biophysica Sinica, 53*(6), 748–757. 10.1093/abbs/gmab043 33891698

[67] Park, J. H., Choi, Y., Lim, C. W., Park, J. M., Yu, S. H., Kim, Y., et al. (2021). Potential therapeutic effect of micrornas in extracellular vesicles from mesenchymal stem cells against SARS-CoV-2. *Cells, 10*(9), 2393. 10.3390/cells10092393 PMC8465096 34572043

[68] Kim, S. Y., Joglekar, M. V., Hardikar, A. A., Phan, T. H., Khanal, D., Tharkar, P., et al. (2019). Placenta stem/stromal cell-derived extracellular vesicles for potential use in lung repair. *Proteomics, 19*(17), e1800166. 10.1002/pmic.201800166 31318160

[69] Yi, X., Wei, X., Lv, H., An, Y., Li, L., Lu, P., et al. (2019). Exosomes derived from microRNA-30b-3p-overexpressing mesenchymal stem cells protect against lipopolysaccharide-induced acute lung injury by inhibiting SAA3. *Experimental Cell Research, 383*(2), 111454. 10.1016/j.yexcr.2019.05.035 31170401

[70] Hu, S., Park, J., Liu, A., Lee, J., Zhang, X., Hao, Q. (2018). Mesenchymal stem cell microvesicles restore protein permeability across primary cultures of Injured Human Lung Microvascular endothelial cells. *Stem Cells Translational Medicine, 7*(8), 615–624. 10.1002/sctm.17-0278 PMC6090509 29737632

[71] Wu, X., Liu, Z., Hu, L., Gu, W., Zhu, L. (2018). Exosomes derived from endothelial progenitor cells ameliorate acute lung injury by transferring miR-126. *Experimental Cell Research, 370*(1), 13–23. 10.1016/j.yexcr.2018.06.003 29883714

[72] Wang, H., Zheng, R., Chen, Q., Shao, J., Yu, J., & Hu, S. (2017) . Mesenchymal stem cells microvesicles stabilize endothelial barrier function partly mediated by hepatocyte growth factor (HGF). *Stem Cell Research & Therapy, 8*(1), 211. 10.1186/s13287-017-0662-7 PMC5623961 28969681

[73] Fang SB, Zhang HY, Meng XC, Wang C, He BX, Peng YQ (2020). Small extracellular vesicles derived from human MSCs prevent allergic airway inflammation via immunomodulation on pulmonary macrophages. Cell Death & Disease.

[74] Potter, D. R., Miyazawa, B. Y., Gibb, S. L., Deng, X., Togaratti, P. P., Croze, R. H., et al. (2018). Mesenchymal stem cell-derived extracellular vesicles attenuate pulmonary vascular permeability and lung injury induced by hemorrhagic shock and trauma. *Journal of Trauma and Acute care Surgery, 84*(2), 245–256. 10.1097/TA.0000000000001744 PMC6378956 29251710

[75] Zhao, R., Wang, L., Wang, T., Xian, P., Wang, H., & Long, Q. (2022). Inhalation of MSC-EVs is a noninvasive strategy for ameliorating acute lung injury. *Journal of Controlled Release: Journal of the Controlled Release Society, 345*, 214–230. 10.1016/j.jconrel.2022.03.025 35307508

[76] Xia, L., Zhang, C., Lv, N., Liang, Z., Ma, T., Cheng, H., et al. (2022). AdMSC-derived exosomes alleviate acute lung injury via transferring mitochondrial component to improve homeostasis of alveolar macrophages. *Theranostics, 12*(6), 2928–2947. 10.7150/thno.69533 PMC8965475 35401830

[77] Ikhlas, S., Usman, A., Kim, D., Cai, D. (2021). Exosomes/microvesicles target SARS-CoV-2 via innate and RNA-induced immunity with PIWI-piRNA system. *Life Science Alliance, 5*(3), e202101240. 10.26508/lsa.202101240 PMC8645330 34862272

[78] Liu, X., Gao, C., Wang, Y., Niu, L., Jiang, S., & Pan, S. (2021). BMSC-Derived Exosomes Ameliorate LPS-Induced Acute Lung Injury by miR-384-5p-Controlled Alveolar Macrophage Autophagy. *Oxidative Medicine and Cellular Longevity*, 9973457. 10.1155/2021/9973457 PMC8216833 34234888

[79] Mao, G. C., Gong, C. C., Wang, Z., Sun, M.X., Pei, Z. P., Meng, W. Q., et al. (2021). BMSC-derived exosomes ameliorate sulfur mustard-induced acute lung injury by regulating the GPRC5A-YAP axis. *Acta Pharmacologica Sinica*, *42*(12), 2082–2093. 10.1038/s41401-021-00625-4 PMC8633287 33654219

[80] Chen, W. X., Zhou, J., Zhou, S. S., Zhang, Y. D., Ji, T. Y., Zhang, X. L., et al. (2020). Microvesicles derived from human Wharton’s jelly mesenchymal stem cells enhance autophagy and ameliorate acute lung injury via delivery of miR-100. *Stem Cell Research & Therapy, 11*(1), 113. 10.1186/s13287-020-01617-7 PMC7071666 32169098

[81] Ibrahim, A. G., Ciullo, A., Li, C., Garcia, G., Peck, K., Miyamoto, K., et al. (2022). Engineered extracellular vesicles antagonize SARS-CoV-2 Infection by inhibiting mTOR signaling. *Biomaterials and Biosystems, 6*, 100042. 10.1016/j.bbiosy.2022.100042 PMC8841010 35187508

[82] Théry, C., Witwer, K. W., Aikawa, E., Alcaraz, M. J., Anderson, J. D., Andriantsitohaina, R. (2018). Minimal information for studies of extracellular vesicles 2018 (MISEV2018): A position statement of the International Society for Extracellular Vesicles and update of the MISEV2014 guidelines. *Journal of Extracellular Vesicles, 7*(1), 1535750. 10.1080/20013078.2018.1535750 10.1080/20013078.2018.1535750 PMC6322352 30637094

[83] Silva J, Garcia V, Rodriguez M, Compte M, Cisneros E, Veguillas P (2012). Analysis of exosome release and its prognostic value in human Colorectal cancer. Genes, Chromosomes & Cancer.

[84] Gurer DC, Akgül B (2023). Noncoding RNAs: A new layer of functional RNAs. Current Pharmaceutical Biotechnology.

[85] Rebelatto, C. L. K., Senegaglia, A. C., Franck, C. L., Daga, D. R., Shigunov, P., Stimamiglio, M. A., et al. (2022). Safety and long-term improvement of mesenchymal stromal cell infusion in critically COVID-19 patients: A randomized clinical trial. *Stem cell Research & Therapy, 13*(1), 122. 10.1186/s13287-022-02796-1 PMC8935270 35313959

[86] Saleh, M., Vaezi, A. A., Aliannejad, R., Sohrabpour, A. A., Kiaei, S. Z. F., Shadnoush, M., et al. (2021). Cell therapy in patients with COVID-19 using Wharton’s jelly mesenchymal stem cells: a phase 1 clinical trial. *Stem cell Research & Therapy, 12*(1), 410. 10.1186/s13287-021-02483-7 PMC8283394 34271988

[87] Sharma, D., & Zhao, F. (2021).Updates on clinical trials evaluating the regenerative potential of allogenic mesenchymal stem cells in COVID-19. *NPJ Regenerative Medicine, 6*(1), 37. 10.1038/s41536-021-00147-x PMC8245638 34193864

[88] Zanirati, G., Provenzi, L., Libermann, L. L., Bizotto, S. C., Ghilardi, I. M., Marinowic, D. R., et al. (2021). Stem cell-based therapy for COVID-19 and ARDS: A systematic review. *NPJ Regenerative Medicine, 6*(1), 73. 10.1038/s41536-021-00181-9 PMC8575895 34750382

[89] Cuevas-Gonzalez, M. V., Garcia-Perez, Á., Gonzalez-Aragon Pineda, Á. E., Espinosa-Cristobal, L. F., Donohue-Cornejo, A., Tovar-Carrillo, K. L., et al. (2021). Stem Cells as a Model of Study of SARS-CoV-2 and COVID-19: A Systematic Review of the Literature. *BioMed Research International*, 9915927. 10.1155/2021/9915927 PMC8390136 34458372

[90] Wang, J., Shi, P., Chen, D., Wang, S., Wang, P., Feng, X., et al. (2021). Research Status of the Safety and Efficacy of Mesenchymal Stem Cells in the Treatment of COVID-19-Related Pneumonia: A Systematic Review and Meta-Analysis. *Stem cells and development, 30*(19), 947–969. 10.1089/scd.2021.0179 34416823

[91] Su, V. Y. F., Lin, C. S., Hung, S. C., Yang, K. Y. (2019). Mesenchymal stem cell-conditioned medium induces Neutrophil apoptosis Associated with inhibition of the NF-κB pathway in Endotoxin-Induced Acute Lung Injury. *International Journal of Molecular Sciences, 20*(9), 2208. 10.3390/ijms20092208 PMC6540353 31060326

[92] Yan, X., Fu, X., Jia, Y., Ma, X., Tao, J., Yang, T., et al. (2019). Nrf2/Keap1/ARE Signaling Mediated an Antioxidative Protection of Human Placental Mesenchymal Stem Cells of Fetal Origin in Alveolar Epithelial Cells. *Oxidative Medicine and Cellular Longevity*, 2654910. 10.1155/2019/2654910 PMC6537011 31217836

[93] Xiao, K., He, W., Guan, W., Hou, F., Yan, P., Xu, J., Zhou, T., Liu, Y., Xie, L., et al. (2020). Mesenchymal stem cells reverse EMT process through blocking the activation of NF-κB and hedgehog pathways in LPS-induced acute lung injury. *Cell Death & Disease, 11*(10), 863. 10.1038/s41419-020-03034-3 PMC7567061 33060560

[94] Jackson, M. V., Krasnodembskaya, A. D. (2017). Analysis of mitochondrial transfer in direct co-cultures of human monocyte-derived macrophages (MDM) and mesenchymal stem cells (MSC). *Bio-Protocol, 7*(9), e2255. 10.21769/BioProtoc.2255 PMC5438594 28534038

[95] Sinha, D., Roy, S., Saha, P., Chatterjee, N., Bishayee, A. (2021). Trends in research on exosomes in cancer progression and anticancer therapy. *Cancers, 13*(2), 326. 10.3390/cancers13020326 PMC7829710 33477340

[96] Askenase, P. W. (2020). COVID-19 therapy with mesenchymal stromal cells (MSC) and convalescent plasma must consider exosome involvement: Do the exosomes in convalescent plasma antagonize the weak immune antibodies? *Journal of Extracellular Vesicles, 10*(1), e12004. 10.1002/jev2.12004 PMC7710130 33304473

[97] Asgharzade, S., Alizadeh, A., Arab, S. (2021). Regenerative medicine approaches in COVID-19 Pneumonia. *Current stem cell Research & Therapy, 16*(6), 647–655. 10.2174/1574888X16999210112205826 33438550

[98] Park, J., Jeong, S., Park, K., Yang, K., Shin, S. (2018). Expression profile of microRNAs following bone marrow-derived mesenchymal stem cell treatment in lipopolysaccharide-induced acute lung injury. *Experimental and Therapeutic Medicine, 15*(6), 5495–5502. 10.3892/etm.2018.6118 PMC5996665 29904430

[99] Li, J., Huang, S., Zhang, J., Feng, C., Gao, D., Yao,B. et al. (2016). Mesenchymal stem cells ameliorate inflammatory cytokine-induced impairment of AT-II cells through a keratinocyte growth factor-dependent PI3K/Akt/mTOR signaling pathway. *Molecular Medicine Reports, 13*(5), 3755–62. 10.3892/mmr.2016.5004 PMC4838139 27035760

[100] Crisostomo, P. R., Markel, T. A., Wang, Y., Meldrum, D. R. (2008). Surgically relevant aspects of stem cell paracrine effects. *Surgery, 143*(5), 577–81. 10.1016/j.surg.2007.10.015 PMC2387075 18436004

[101] Perreau, M., Suffiotti, M., Marques-Vidal, P., Wiedemann, A., Levy, Y., et al. (2021). The cytokines HGF and CXCL13 predict the severity and the mortality in COVID-19 patients. *Nature Communications, 12*(1), 4888. 10.1038/s41467-021-25191-5 PMC8352963 34373466

[102] Vishnupriya, M., Naveenkumar, M., Manjima, K., Sooryasree, N. V., Saranya, T., Ramya, S., et al. (2021). Post-COVID pulmonary fibrosis: Therapeutic efficacy using with mesenchymal stem cells - how the lung heals. *European Review for Medical and Pharmacological Sciences, 25*(6), 2748–2751. 10.26355/eurrev_202103_25438 33829461

[103] Adas, G., Cukurova, Z., Yasar, K. K., Yilmaz, R., Isiksacan, N., Kasapoglu, P., et al. (2021). The systematic effect of mesenchymal stem cell therapy in critical COVID-19 patients: A prospective double controlled trial. *Cell Transplantation, 30*, 9636897211024942. 10.1177/09636897211024942 PMC8243094 34180719

[104] Lu, R. X. Z., Lai, B. F. L., Rafatian, N., Gustafson, D., Campbell, S. B., Banerjee, A. (2022). Vasculature-on-a-chip platform with innate immunity enables identification of angiopoietin-1 derived peptide as a therapeutic for SARS-CoV-2 induced inflammation. 10.1039/d1lc00817j PMC9207819 35142777

[105] Witwer, K. W., Goberdhan, D. C., O'Driscoll, L., Théry, C., Welsh, J. A., Blenkiron, C., et al. (2021) Updating MISEV: Evolving the minimal requirements for studies of extracellular vesicles. *Journal of Extracellular Vesicles, 10*(14), e12182. 10.1002/jev2.12182 PMC8710080 34953156

[106] Khalaj, K., Figueira, R. L., Antounians, L., Lauriti, G., Zani, A. (2020). Systematic review of extracellular vesicle-based treatments for lung injury: Are EVs a potential therapy for COVID-19? *Journal of Extracellular Vesicles, 9*(1), 1795365. 10.1080/20013078.2020.1795365 PMC7481829 32944185

[107] Weiss, D. J., Lim, S. K., Rohde, E., Witwer, K. W., Giebel, B. (2020). Weiss Response to Sengupta. *Stem Cells and Development, 29*(24), 1533–1534. 10.1089/scd.2020.0095 10.1089/scd.2020.0114 33301389

[108] Reiner, A. T., Witwer, K. W., van Balkom, B. W. M., de Beer, J. de, Brodie, C., Corteling, R. L. (2017) Concise Review: Developing best-practice models for the therapeutic use of Extracellular vesicles. *Stem Cells Translational Medicine, 6*(8), 1730–1739. 10.1002/sctm.17-0055 PMC5689784 28714557

[109] Inal, J. M. (2020). Decoy ACE2-expressing extracellular vesicles that competitively bind SARS-CoV-2 as a possible COVID-19 therapy. *Clinical Science (London, England: 1979), 134*(12), 1301–1304. 10.1042/CS20200623 PMC7298154 32542396

[110] Scott, T. A., Supramaniam, A., Idris, A., Cardoso, A. A., Shrivastava, S., Kelly, G., et al. (2022). Engineered extracellular vesicles directed to the spike protein inhibit SARS-CoV-2. *Molecular Therapy Methods & Clinical Development, 24, 355*–*366*. 10.1016/j.omtm.2022.01.015 PMC8806709 35127966

[111] Börger, V., Weiss, D. J., Anderson, J. D., Borràs, F. E., Bussolati, B., Carter, D. R. F., et al. (2020). International Society for Extracellular Vesicles and International Society for Cell and Gene Therapy statement on extracellular vesicles from mesenchymal stromal cells and other cells: considerations for potential therapeutic agents to suppress coronavirus disease-19. *Cytotherapy, 22*(9), 482–485. 10.1016/j.jcyt.2020.05.002 PMC7229942 32425691

[112] Chance, T. C., Rathbone, C. R., Kamucheka, R. M., Peltier, G. C., Cap, A. P., Bynum, J. A. (2019) The effects of cell type and culture condition on the procoagulant activity of human mesenchymal stromal cell-derived extracellular vesicles. *The Journal of Trauma and Acute Care Surgery, 87*, S74–S82. 10.1097/TA.0000000000002225 31246910

[113] Silachev, D. N., Goryunov, K. V., Shpilyuk, M. A., et al. (2019). Effect of MSCs and MSC-Derived extracellular vesicles on human blood coagulation. *Cells, 8*(3), 258. 10.3390/cells8030258 PMC6468445 30893822

[114] Corritori S, Savchuk N, Pauza CD (2022). Risk/Benefit profiles of currently approved oral antivirals for treatment of COVID-19: Similarities and differences. COVID.

[115] Alipour S, Mahmoudi L, Ahmadi F (2022). Pulmonary drug delivery: An effective and convenient delivery route to combat COVID-19. Drug Delivery and Translational Research.

[116] ClinicalTrials.gov [Internet]. Bethesda (MD): National Library of Medicine (US). 2000 Feb 29. Identifier NCT04491240, Evaluation of Safety and Efficiency of Method of Exosome Inhalation in SARS-CoV-2 Associated Pneumonia; 2020 July 6 [cited 2022 Feb 27]. Available from: https://clinicaltrials.gov/study/NCT04491240

[117] Marchiano, S., Hsiang, T. Y., Khanna, A., Higashi, T., Whitmore, L. S., Bargehr, J., et al. (2021). SARS-CoV-2 infects human pluripotent stem cell-derived cardiomyocytes, impairing electrical and mechanical function. *Stem cell Reports, 16*(3), 478–492. 10.1016/j.stemcr.2021.02.008 PMC7881699 33657418

[118] Guzik, T. J., Mohiddin, S. A., Dimarco, A., Patel, V., Savvatis, K., Marelli-Berg, F. M., et al. (2020). COVID-19 and the cardiovascular system: Implications for risk assessment, diagnosis, and treatment options. *Cardiovascular Research, 116*(10), 1666–1687. 10.1093/cvr/cvaa106 PMC7197627 32352535

[119] Ragni, E., Banfi, F., Barilani, M., Cherubini, A., Parazzi, V., Larghi, P., et al. (2017). Extracellular vesicle-shuttled mRNA in mesenchymal stem cell communication. *Stem Cells, 35*(4), 1093–1105. 10.1002/stem.2557 28164431

[120] Kanada, M., Bachmann, M. H., Hardy, J. W., Frimannson, D. O., Bronsart, L., Wang, A., et al. (2015). Differential fates of biomolecules delivered to target cells via extracellular vesicles. *Proceedings of the National Academy of Sciences of the United States of America, 112*(12), E1433–42. 10.1073/pnas.1418401112 PMC4378439 25713383

