# Supplementary material for: Stem Cell Extracellular Vesicles as Anti-SARS-CoV-2 Immunomodulatory Therapeutics: A Systematic Review of Clinical and Preclinical Studies
Source: Stem Cell Rev Rep. 2024 Feb 23;20(4):900–30. doi: 10.1007/s12015-023-10675-2 (PMC11087360; doi:10.1007/s12015-023-10675-2)
Supplement: Supplementary file 1 — Supplementary Material 1 (docx 22.9 KB) [file 12015_2023_10675_MOESM1_ESM.docx]

| Research Question | Stem cell EVs as therapeutic agents against COVID-19 |
| --- | --- |
| Databases | Pubmed, Scopus, Web of science, Cochrane Central Register of Controlled Trials |
| Population | Symptomatic or asymptomatic confirmed SARS-CoV-2 patients (by nucleic acid amplification testing, antibody assay, or antigen testing)  or ARDS /lung injury/ Pneumonia animal models |
| Intervention | Stem cells derived EVs |
| Comparator | Conventional therapies for COVID-19 or placebo |
| Outcome | 1. Survival rate 2. Severity of clinical symptoms (fever, cough, shortness of breath, chest pain, ICU clinical status, SOFA score etc.) 3. Change in oxygenation levels (e.g., PaO2/FiO2 ratio) 4. Presence and size/degree of pulmonary lesions /lung injury 5. Circulating levels of immune cells ( white blood cells, lymphocytes, neutrophils, macrophages, regulatory dendritic cells, NK cells) 6. Pro-inflammatory cytokines, (IL-6, TNF-α, IFN-γ, etc.) 7. Anti-inflammatory cytokines (IL-10, TGF-β, etc.) 8. Inflammatory markers (C-reactive protein, Ferritin, D-dimer, etc.) 9. Adverse events arising from MSC/EVs administration (proliferation, apoptosis, migration, tumorigenesis, thromboembolism, ectopic tissue formation, angieogensis, etc.). 10. Lung protein permeability 11. miRNA specific expression levels |
| Inclusion criteria | 1. Studies that uses stem cells EVs to treat COVID-19/ ARDS/ Lung injury 2. Stem cell derived EVs from any tissue source (e.g., bone marrow, adipose, umbilical cord, dental pulp, placenta, etc.). 3. Tissues from which SC/secretome are obtained may be syngeneic, allogeneic, or xenogeneic. 4. All routes of SC-secretome administration will be considered (intravenous injection, aerosol inhalation, intramuscular injection, etc.). 5. SCs/secretome may be administered along with other therapeutic agents (antivirals, anti-cytokine drugs, immunomodulatory agents, etc.). 6. Studies mentioning EVs isolation methods (Ultracentrifugation, Polymer precipitation (PEG), magnetic beads, ultrafiltration or commercial kits) 7. Studies mentioning characterization methods for EVs (TEM, Dynamic light scaterring, Western blot, Nano particle tracking analysis ) |
| Exclusion criteria | - 1. Studies in which only non-Stem Cell-based therapeutics are administered to treat COVID-19, such as non-Stem cells, antivirals, immunomodulatory drugs combination therapies, and anti-cytokine drugs   2. Review articles, meta-analysis, comments, notes, book chapters or surveys and theses, conference proceedings and editorals and bibliographies will be excluded commercial documentations .   3. Articles in languages other than English   (4) Duplicates or articles that were found in common among the three selected search engines. |
| Keywords | Stromal cells, Mesenchymal stem cells, Adipose stem cells, Umblical cord stem cells, Extracellular vesicles, Exosomes, Microvesicles, Coronavirus disease 2019, COVID-19, Severe acute respiratory syndrome coronavirus 2, SARS-CoV-2, Acute respiratory distress syndrome, ARDS, Pneumonia |
| Search | 1. (exosome* OR extracellular vesicle* OR microvesicle*). 2. (COVID-19 OR SARS-CoV-2 OR lung*OR respiratory* OR pulmonary*). 3. 1 AND 2 |
