# Supplementary material for: Stem Cell Extracellular Vesicles as Anti-SARS-CoV-2 Immunomodulatory Therapeutics: A Systematic Review of Clinical and Preclinical Studies
Source: Stem Cell Rev Rep. 2024 Feb 23;20(4):900–30. doi: 10.1007/s12015-023-10675-2 (PMC11087360; doi:10.1007/s12015-023-10675-2)
Supplement: Supplementary file 2 — Supplementary Material 2 (docx 26.2 KB) [file 12015_2023_10675_MOESM2_ESM.docx]

| **List of excluded studies with reasons for exclusion** | | |
| --- | --- | --- |
| **Article** | **Reason for exclusion** |  |
| Schultz IC, Bertoni APS, Wink MR. Mesenchymal Stem Cell-Derived Extracellular Vesicles Carrying miRNA as a Potential Multi Target Therapy to COVID-19: an In Silico Analysis. Stem cell Rev reports. 2021 Apr;17(2):341–56. | Study conducted as bioinformatics analysis model |  |
| Zhao M, Liu S, Wang C, Wang Y, Wan M, Liu F, et al. Mesenchymal Stem Cell-Derived Extracellular Vesicles Attenuate Mitochondrial Damage and Inflammation by Stabilizing Mitochondrial DNA. ACS Nano. 2020/12/29. 2021;15(1):1519–38. | Study conducted on AKI model |  |
| Dong L, Wang Y, Zheng T, Pu Y, Ma Y, Qi X, et al. Hypoxic hUCMSC-derived extracellular vesicles attenuate allergic airway inflammation and airway remodeling in chronic asthma mice. Stem Cell Res Ther. 2021 Jan;12(1):4. | Study conducted on an asthma model |  |
| Zhang E, Geng X, Shan S, Li P, Li S, Li W, et al. Exosomes derived from bone marrow mesenchymal stem cells reverse epithelial-mesenchymal transition potentially via attenuating Wnt/β-catenin signaling to alleviate silica-induced pulmonary fibrosis. Toxicol Mech Methods. 2021/07/07. 2021;31(9):655–66. | Study conducted on a pulmonary fibrosis model |  |
| Elashiry M, Elsayed R, Elashiry MM, Rashid MH, Ara R, Arbab AS, et al. Proteomic Characterization, Biodistribution, and Functional Studies of Immune-Therapeutic Exosomes: Implications for Inflammatory Lung Diseases [Internet]. Vol. 12, Frontiers in Immunology. 2021. Available from: https://www.frontiersin.org/article/10.3389/fimmu.2021.636222 | Study not reporting on EV treatment |  |
| Rosell A, Havervall S, von Meijenfeldt F, Hisada Y, Aguilera K, Grover SP, et al. Patients With COVID-19 Have Elevated Levels of Circulating Extracellular Vesicle Tissue Factor Activity That Is Associated With Severity and Mortality-Brief Report. Arter Thromb Vasc Biol. 2020/12/04. 2021;41(2):878–82. | Study not reporting on EV treatment |  |
| Meidert AS, Hermann S, Brandes F, Kirchner B, Buschmann D, Billaud JN, et al. Extracellular Vesicle Associated miRNAs Regulate Signaling Pathways Involved in COVID-19 Pneumonia and the Progression to Severe Acute Respiratory Corona Virus-2 Syndrome. Front Immunol. 2021/12/28. 2021;12:784028. | Study not reporting on EV treatment |  |
| Nazerian Y, Vakili K, Ebrahimi A, Niknejad H. Developing Cytokine Storm-Sensitive Therapeutic Strategy in COVID-19 Using 8P9R Chimeric Peptide and Soluble ACE2. Front Cell Dev Biol. 2021 Sep 3;9:717587. doi: 10.3389/fcell.2021.717587. PMID: 34540833; PMCID: PMC8446510. | Study not reporting on EV treatment |  |
| Dinh P-UC, Paudel D, Brochu H, Popowski KD, Gracieux MC, Cores J, et al. Inhalation of lung spheroid cell secretome and exosomes promotes lung repair in pulmonary fibrosis. Nat Commun [Internet]. 2020;11(1):1064. Available from: https://doi.org/10.1038/s41467-020-14344-7 | Study conducted on a pulmonary fibrosis model |  |
| Zhang C, Guo F, Chang M, Zhou Z, Yi L, Gao C, et al. Exosome-delivered syndecan-1 rescues acute lung injury via a FAK/p190RhoGAP/RhoA/ROCK/NF-κB signaling axis and glycocalyx enhancement. Exp Cell Res. 2019 Nov;384(1):111596. | Study not reporting on EV treatment |  |
| Mansouri N, Willis GR, Fernandez-Gonzalez A, Reis M, Nassiri S, Mitsialis SA, et al. Mesenchymal stromal cell exosomes prevent and revert experimental pulmonary fibrosis through modulation of monocyte phenotypes. JCI insight. 2019 Nov;4(21). | Study conducted on a pulmonary fibrosis model |  |
| Loy H, Kuok DIT, Hui KPY, Choi MHL, Yuen W, Nicholls JM, et al. Therapeutic Implications of Human Umbilical Cord Mesenchymal Stromal Cells in Attenuating Influenza A(H5N1) Virus-Associated Acute Lung Injury. J Infect Dis. 2019 Jan;219(2):186–96. | Study conducted on an H5N1 model |  |
| Li Q-C, Liang Y, Su Z-B. Prophylactic treatment with MSC-derived exosomes attenuates traumatic acute lung injury in rats. Am J Physiol Lung Cell Mol Physiol. 2019 Jun;316(6):L1107–17. | Study not reporting on EV treatment |  |
| Sun L, Zhu M, Feng W, Lin Y, Yin J, Jin J, et al. Exosomal miRNA Let-7 from Menstrual Blood-Derived Endometrial Stem Cells Alleviates Pulmonary Fibrosis through Regulating Mitochondrial DNA Damage. Oxid Med Cell Longev. 2019; 2019:4506303. | Study conducted on a sepsis model |  |
| Sun A, Lai Z, Zhao M, Mu L, Hu X. Native nanodiscs from blood inhibit pulmonary fibrosis. Biomaterials. 2019 Feb; 192:51–61. | Study conducted on a pulmonary fibrosis model |  |
| Bandeira E, Oliveira H, Silva JD, Menna-Barreto RFS, Takyia CM, Suk JS, et al. Therapeutic effects of adipose-tissue-derived mesenchymal stromal cells and their extracellular vesicles in experimental silicosis. Respir Res. 2018 May;19(1):104. | Study conducted on an experimental silicosis model |  |
| Tan JL, Lau SN, Leaw B, Nguyen HPT, Salamonsen LA, Saad MI, et al. Amnion Epithelial Cell-Derived Exosomes Restrict Lung Injury and Enhance Endogenous Lung Repair. Stem Cells Transl Med. 2018 Feb;7(2):180–96. | Study not reporting on EV treatment |  |

**Abbreviations**

**AKI:** Acute kidney injury

**EV**: Extracellular vesicle

**COVID-19:** Coronavirus disease-2019
