# Supplementary material for: Stem Cell Extracellular Vesicles as Anti-SARS-CoV-2 Immunomodulatory Therapeutics: A Systematic Review of Clinical and Preclinical Studies
Source: Stem Cell Rev Rep. 2024 Feb 23;20(4):900–30. doi: 10.1007/s12015-023-10675-2 (PMC11087360; doi:10.1007/s12015-023-10675-2)
Supplement: Supplementary file 3 — Supplementary Material 3 (docx 26.4 KB) [file 12015_2023_10675_MOESM3_ESM.docx]

**SYRCLE’s risk of bias tool**

|  | **Selection bias** | | | **Performance bias** | | **Detection bias** | | **Attrition bias** | **Reporting bias** | **Other** | | | | |
| --- | --- | --- | --- | --- | --- | --- | --- | --- | --- | --- | --- | --- | --- | --- |
| **Study** | Sequence generation | Baseline characteristics | Allocation concealment | Random housing | Blinding | Random outcome assessment | Blinding | Incomplete outcome data | Selective outcome reporting | Free of contamination | Free of inappropriate influence of funders | Free of unit of analysis errors | Design-specific risk of bias | New animals added to replace drop-outs |
| **Kaspi 2021** | unclear | yes | yes | yes | yes | unclear | yes | yes | yes | yes | yes | yes | no | unclear |
| **Silva 2021** | unclear | yes | unclear | unclear | unclear | unclear | unclear | yes | yes | yes | no | yes | no | unclear |
| **Cloer 2021** | unclear | yes | unclear | unclear | unclear | unclear | unclear | yes | yes | unclear | yes | yes | no | unclear |
| **Shi 2021** | no | yes | no | no | no | unclear | unclear | yes | yes | yes | yes | yes | no | no |
| **Mizuta 2020** | unclear | yes | unclear | unclear | unclear | unclear | yes | unclear | yes | unclear | yes | yes | no | unclear |
| **Deng 2020** | unclear | yes | unclear | unclear | unclear | unclear | yes | yes | yes | unclear | unclear | yes | no | no |
| **Xu 2021** | unclear | yes | unclear | unclear | unclear | unclear | yes | yes | yes | yes | yes | yes | no | no |
| **Wei 2020** | unclear | yes | unclear | unclear | unclear | unclear | yes | unclear | yes | unclear | yes | yes | no | unclear |
| **Sui 2021** | unclear | yes | unclear | unclear | unclear | unclear | yes | unclear | yes | unclear | yes | yes | no | unclear |
| **Tian 2021** | unclear | yes | unclear | unclear | unclear | unclear | yes | unclear | yes | unclear | yes | yes | no | unclear |
| **Wang 2020** | unclear | yes | unclear | unclear | unclear | unclear | yes | yes | yes | unclear | yes | no | no | unclear |
| **Zheng 2021** | unclear | yes | unclear | unclear | yes | unclear | yes | unclear | yes | unclear | yes | unclear | no | unclear |
| **Yi 2019** | unclear | yes | unclear | unclear | unclear | unclear | yes | unclear | yes | unclear | yes | yes | no | no |
| **Chen 2019** | unclear | yes | unclear | unclear | unclear | unclear | yes | unclear | yes | unclear | yes | yes | no | unclear |
| **Xu 2019** | unclear | yes | unclear | unclear | unclear | unclear | yes | unclear | yes | unclear | yes | yes | no | unclear |
| **Varkouhi 2019** | unclear | yes | unclear | unclear | yes | yes | yes | yes | yes | unclear | yes | yes | no | yes |
| **Zhou 2019** | unclear | yes | unclear | unclear | yes | yes | yes | unclear | yes | yes | yes | no | no | unclear |
| **Liua 2019** | unclear | yes | unclear | unclear | unclear | unclear | unclear | unclear | yes | unclear | yes | no | no | unclear |
| **Wu 2018** | unclear | yes | unclear | unclear | unclear | unclear | yes | unclear | yes | yes | yes | yes | no | unclear |
| **Tang 2017** | unclear | yes | unclear | unclear | unclear | yes | yes | unclear | yes | unclear | yes | no | no | unclear |
| **Li 2015** | unclear | no | unclear | unclear | unclear | unclear | unclear | unclear | yes | unclear | yes | no | no | unclear |
| **Monsel 2015** | unclear | yes | unclear | unclear | unclear | unclear | unclear | unclear | yes | unclear | unclear | no | no | unclear |
| **Zhu 2014** | unclear | yes | unclear | unclear | unclear | unclear | unclear | unclear | yes | unclear | no | no | no | unclear |
| **Fang 2020** | unclear | yes | unclear | unclear | unclear | unclear | unclear | unclear | yes | yes | yes | yes | no | unclear |
| **Huang 2019** | unclear | yes | unclear | unclear | unclear | unclear | yes | unclear | yes | unclear | yes | yes | no | unclear |
| **Silva 2019** | yes | yes | unclear | no | yes | yes | yes | unclear | yes | yes | yes | no | no | unclear |
| **Potter 2018** | unclear | yes | unclear | unclear | unclear | yes | yes | unclear | yes | unclear | yes | yes | no | unclear |
| **Khatri 2018** | unclear | yes | unclear | unclear | unclear | unclear | yes | yes | yes | unclear | yes | yes | no | yes |
| **Morrison 2017** | unclear | yes | unclear | unclear | unclear | unclear | unclear | unclear | yes | unclear | unclear | no | no | unclear |
| **Gao 2020** | unclear | yes | unclear | unclear | unclear | unclear | unclear | yes | yes | unclear | yes | yes | no | yes |
| **Yu 2020** | unclear | yes | unclear | unclear | unclear | yes | unclear | unclear | yes | unclear | yes | yes | no | unclear |
| **Zhao 2022** | unclear | yes | unclear | yes | yes | yes | yes | yes | yes | yes | yes | yes | no | yes |
| **Xia 2022** | unclear | yes | unclear | yes | unclear | unclear | yes | yes | yes | yes | yes | no | no | yes |

**Modified SYRCLE’s risk of bias tool.**

| **Study** | **Sequence generation** | **allocation concealment** | **experimental conditions** | **Blinding** | **Incomplete outcome data** | **exposure characterization** | **outcome assessment** | **Selective outcome reporting** |
| --- | --- | --- | --- | --- | --- | --- | --- | --- |
| **Park 2021** | yes | yes | yes | yes | yes | yes | yes | yes |
| **Li 2020** | unclear | unclear | yes | unclear | yes | yes | yes | yes |
| **Kim 2019** | yes | yes | yes | yes | yes | yes | yes | yes |
| **Park 2019** | yes | yes | yes | yes | yes | yes | yes | yes |
| **Hu 2018** | yes | yes | yes | yes | yes | yes | yes | yes |
| **Wang 2017** | yes | yes | yes | yes | yes | yes | yes | yes |

**ROBINS-I risk of bias tool.**

| **Study** | **Bias due to confounding** | **Bias in selection of participants into the study** | **Bias in classification of interventions** | **Bias due to deviations from intended interventions** | **Bias due to missing data** | **Bias in measurement of outcomes** | **Bias in selection of the reported result** | **Overall Bias** |
| --- | --- | --- | --- | --- | --- | --- | --- | --- |
| **Sengupta 2020** | Low | Low | Low | Moderate | Low | Moderate | Low | Low |
| **Zhu 2022** | Moderate | Low | Low | Low | Low | Low | Low | Moderate |
| **Mitrani 2021** | Moderate | Low | Low | Low | Low | Moderate | Low | Moderate |
| **Bellioa 2021** | Low | Low | Low | Low | Low | Low | Low | Low |

**ROB 2 risk of bias tool.**

| **Study** | **Randomization process** | **Deviations from intended interventions** | **Mising outcome data** | **Measurement of the outcome** | **Selection of the reported result** | **Overall Bias** |
| --- | --- | --- | --- | --- | --- | --- |
| **Fathi‑Kazerooni 2022** | Low | Low | Low | Low | Low | Low |
