# Supplementary material for: Stem Cell Extracellular Vesicles as Anti-SARS-CoV-2 Immunomodulatory Therapeutics: A Systematic Review of Clinical and Preclinical Studies
Source: Stem Cell Rev Rep. 2024 Feb 23;20(4):900–30. doi: 10.1007/s12015-023-10675-2 (PMC11087360; doi:10.1007/s12015-023-10675-2)
Supplement: Supplementary file 4 — Supplementary Material 4 (docx 11.3 KB) [file 12015_2023_10675_MOESM4_ESM.docx]

**Summary of the confidence rating of outcomes (CERQual Qualitative Evidence Profile Table).**

| **Outcomes** | **Studies contributing to the**  **outcomes** | **Domain 1: the methodological limitations** | **Domain 2: the relevance** | **Domain 3: the coherence** | **Domain 4: the adequacy of the data** | **Overall CERQual**  **rating for assessment of confidence** |
| --- | --- | --- | --- | --- | --- | --- |
| **Inflammatory response** | Zhu 2022,Xia 2022, Zhao 2022, F‑Kazerooni 2022, Mitrani 2021, Mitrani 2021, Bellio 2021, Park 2021, Kaspi 2021, Silva 2021, Cloer 2021, Shi 2021, Xu 2021, Tian 2021, Zheng 2021, Deng 2020, Wei 2020, Li 2020, Sengupta 2020, Fang 2020, Wang 2020, Gao 2020, Yu 2020, Sui 2021, Huang 2019, Silva 2019, Kim 2019, Yi 2019, Chen 2019, Liu 2019, Zhou 2019, Xu 2019, Varkouhi 2019, Park 2019, Potter 2018, Khatri 2018, Wu 2018, Tang 2017, Wang 2017, Morrison 2017, Monsel 2015, Li 2015, Zhu 2014. | There were methodological  limitations in the domains  important to internal validity in primary studies, especially blinding for assessors and grouping randomization and concealment. | Generalizability to clinical trials could be feasible with minor limitations given by practical assessment instruments. | All the studies reported significant inflammatory responses. However, one study (Hu 2018) found minimal effect on inflammatory response, and it was explained by the highly used concentrations of cytomix to induce ARDS, which in turn affected the MSC therapeutic potency. In another study (Mizuta 2020), the response was unclear. | Most of the included studies reported outcomes on inflammatory response. Most studies evaluated both pro-inflammation and anti-inflammatory cytokines (CRP, ferritin, D-dimer, and interleukins), neutrophil infiltration, total cell count, lymphocyte count and M2 macrophage polarization. | ⊕⊕⊕⊕  High |
| **Recover lung injury in alveolar epithelium** | Zhu 2022, Xia 2022, Zhao 2022, F‑Kazerooni 2022, Mitrani 2021, Mitrani 2021, Bellio 2021, Park et al 2021, Kaspi et al 2021, Silva 2021, Cloer 2021, Shi 2021, Xu 2021, Sui 2021, Tian 2021, Zheng 2021, Fang 2020, Sengupta 2020, Mizuta 2020, Deng 2020, Wang 2020, Gao 2020, Yu 2020, Wei 2020, Li 2020, Silva 2019, Kim 2019, Yi 2019, Chen 2019, Huang 2019, Xu 2019, Varkouhi 2019, Zhou 2019, Park 2019, Liu 2019, Wu 2018, Potter 2018, Hu 2018, Khatri 2018, Morrison 2017, Wang 2017, Tang 2017, Monsel 2015, Zhu 2014. | There were methodological  limitations in the domains  important to internal validity in primary studies, especially blinding for assessors and grouping randomization and concealment. | Transformation to clinical trials is mostly limited by practical and feasible assessment instruments. | All 44 studies found a significantly decreased endothelial damage and apoptosis, oxidative stress, vascular hyperpermeability, and/or stimulation of surfactant production and re-expansion of alveolus. Only one study (Li 2015) didn’t measure lung recovery. | Almost all the included studies reported outcomes on lung injury in alveolar epithelium. Most of these papers evaluated apoptosis of alveolar epithelium, lung injury and SOFA score, wet/dry ratio, PaO2/ FiO2 ratio, oxygen saturation, vascular hyper-permeability, stimulation of surfactant production, re-expansion of alveolus. | ⊕⊕⊕⊕  High |
| **Improve respiratory function in pulmonary endothelium** | Zhu 2022, Xia 2022, Zhao 2022, F‑Kazerooni 2022, Park et al 2021, Kaspi et al 2021, Shi 2021, Mizuta 2020, Silva 2021, Cloer 2021, Xu 2021, Mitrani 2021, Mitrani 2021, Sengupta 2020, Wang 2020, Yu 2020, Yi 2019, Xu 2019, Varkouhi 2019, Silva 2019, Chen 2019, Zhou 2019, Park 2019, Huang 2019, Wu 2018, Potter 2018, Hu 2018, Khatri 2018, Wang 2017, Tang 2017, Morrison 2017, Li 2015, Monsel 2015, Zhu 2014. | There were methodological  limitations in the domains  important to internal validity in primary studies, especially blinding for assessors and grouping randomization and concealment. | Transformation to clinical trials is mostly limited by practical and feasible assessment instruments. | 35 studies showed a significant restoration of junction proteins and barrier integrity, microvascular permeability repair and respiratory function improvement as increased respiratory rate, minute ventilation, clear chest x-ray and bronchoalveolar lavage fluid content. | 35 out of 45 studies included reported outcomes on respiratory function in pulmonary endothelium. Most of these papers evaluated respiratory function improvement, restoration of junction proteins and microvascular permeability repair and bronchoalveolar lavage fluid content. | ⊕⊕⊕Ο  Moderate |
| **Reduced fibroblasts** | Park 2021, Kaspi 2021, Tian 2021, Silva 2019, Gao 2020, Wang 2020, Yu 2020, Chen 2019, Zhou 2019, Hu 2018, Potter 2018, Wu 2018, Monsel 2015, Zhu 2014. | There were methodological  limitations in the domains  important to internal validity in primary studies, especially  detection bias, reporting bias  and performance bias. | Transformation to clinical trials is mostly limited by practical and feasible assessment instruments. | Moderate concern about coherence since 14 studies found a significant reduction in fibrin production. | 14 out of 45 studies reported outcomes on fibroblasts and evaluated fibrin production. | ⊕ΟΟΟ  Very low |
| **Increase survival rate** | Xia 2022, Shi 2021, Mizuta 2020, Deng 2020, Wei 2020, Sengupta 2020, Silva 2019, Varkouhi 2019, Park 2019, Hu 2018, Gao 2017, Wang 2017, Tang 2017, Li 2015, Monsel 2015. | There were methodological  limitations in the domains  important to internal validity in three studies, especially  blinding for assessors and  grouping randomization and  concealment. | Generalizability to clinical trials is limited by indirect reflection for survival in humans. | Moderate concern about coherence since 15 studies found a significant lengthening in survival rates. | 15 out of 45 studies included reported outcomes on survival rate. | ⊕ΟΟΟ  Very low |
